# Long-chain saturated fatty acids in breast milk are associated with the pathogenesis of atopic dermatitis via induction of inflammatory ILC3s

**DOI:** 10.1038/s41598-021-92282-0

**Published:** 2021-06-23

**Authors:** Weng Sheng Kong, Naohiro Tsuyama, Hiroko Inoue, Yun Guo, Sho Mokuda, Asako Nobukiyo, Nobuhiro Nakatani, Fumiya Yamaide, Taiji Nakano, Yoichi Kohno, Kazutaka Ikeda, Yumiko Nakanishi, Hiroshi Ohno, Makoto Arita, Naoki Shimojo, Masamoto Kanno

**Affiliations:** 1 Department of Immunology, Graduate School of Biomedical and Health Sciences, Hiroshima University, 1-2-3, kasumi, Minami-ku, Hiroshima, 734-8551 Japan; 2 Analytical Molecular Medicine and Devices Laboratory, Hiroshima University, Hiroshima, Japan; 3 Department of Clinical Immunology and Rheumatology, Graduate School of Biomedical and Health Sciences, Hiroshima University, Hiroshima, Japan; 4 Natural Science Centre for Basic Research and Development, Hiroshima University, Hiroshima, Japan; 5 Technical Center, Hiroshima University, Hiroshima, Japan; 6 Department of Pediatrics, Graduate School of Medicine, Chiba University, Chiba, Japan; 7 Laboratory for Metabolomics, RIKEN Center for Integrative Medical Sciences (IMS), Yokohama, Japan; 8 Laboratory for Intestinal Ecosystem, RIKEN Center for Integrative Medical Sciences (IMS), Yokohama, Japan; 9 Intestinal Microbiota Project, Kanagawa Institute of Industrial Science and Technology, Kawasaki, Japan; 10 Immunobiology Laboratory, Graduate School of Medical Life Science, Yokohama City University, Yokohama, Japan; 11 AMED-SENTAN, Tokyo, Japan; 12 AMED-CREST Japan Agency for Medical Research and Development, Tokyo, Japan; 13 Present Address: Department of Radiation Life Sciences, Fukushima Medical University, Fukushima, Japan; 14 Present Address: Chiba Rosai Hospital, Chiba, Japan; 15 Present Address: Laboratory of Medical Omics Research, Kazusa DNA Research Institute, Chiba, Japan; 16 Present Address: Center for Preventive Medicine, Chiba University, Chiba, Japan

**Keywords:** Innate lymphoid cells, Atopic dermatitis, Experimental models of disease

## Abstract

Breastfeeding influences the immune system development in infants and may even affect various immunological responses later in life. Breast milk provides a rich source of early nutrition for infant growth and development. However, the presence of certain compounds in breast milk, related to an unhealthy lifestyle or the diet of lactating mothers, may negatively impact infants. Based on a cohort study of atopic dermatitis (AD), we find the presence of damage-associated molecular patterns (DAMPs) activity in the mother’s milk. By non-targeted metabolomic analysis, we identify the long-chain saturated fatty acids (LCSFA) as a biomarker DAMPs (+) breast milk samples. Similarly, a mouse model in which breastfed offspring are fed milk high in LCSFA show AD onset later in life. We prove that LCSFA are a type of damage-associated molecular patterns, which initiate a series of inflammatory events in the gut involving type 3 innate lymphoid cells (ILC3s). A remarkable increase in inflammatory ILC3s is observed in the gut, and the migration of these ILC3s to the skin may be potential triggers of AD. Gene expression analysis of ILC3s isolated from the gut reveal upregulation of genes that increase ILC3s and chemokines/chemokine receptors, which may play a role in ILC migration to the skin. Even in the absence of adaptive immunity, *Rag1* knockout mice fed a high-LCSFA milk diet develop eczema, accompanied by increased gut ILC3s. We also present that gut microbiota of AD-prone PA milk-fed mice is different from non-AD OA/ND milk-fed mice. Here, we propose that early exposure to LCSFAs in infants may affect the balance of intestinal innate immunity, inducing a highly inflammatory environment with the proliferation of ILC3s and production of interleukin-17 and interleukin-22, these factors may be potential triggers or worsening factors of AD.

## Introduction

Atopic dermatitis (AD) or eczema is a chronic inflammatory itchy disease which starts early in life and has a worldwide prevalence, commonly affects more than one in ten children in developed countries^1,2^. AD is a complex disorder with genetics, barrier function, immunity, and environmental factors all playing key roles. Many of these pathological mechanisms interact synergistically to promote and progress AD^3–5^. Despite considerable advances in the knowledge and understanding of the various factors involved for pathogenesis, there are still many aspects of this complex process that remains poorly understood.

Breast milk not only serves as an optimum food for infants in the early postnatal period and provides immunologic protection against many infections but may also influence the development of the newborn immune system^6–9^. While the nutritional benefits are clear, there is some doubt regarding the effects of breastfeeding in allergies and AD. Epidemiology studies have raised concerns with breastfeeding and risk of allergies and AD^10–13^. Some have suggested a protective effect^14–16^, while other studies reported either no association^17,18^ or a detrimental effect^19,20^. Recent meta-analysis described as “inconclusive evidence” on the association between breastfeeding and allergy in children and mothers^18^. More recently, influence of dietary/breast milk fatty acid on atopic dermatitis was reported as “no association”^21^, which described mainly n-3 and n-6 long-chain polyunsaturated fatty acid (LC-PUFA). These pro/con claims are difficult to prove, however, because immune system dysregulation is multifactorial in origin and may be asymptomatic for several years after weaning. The early positive influences of human breast milk may be a bulwark against chronic disease in later life.

Concerning about the adaptive immunity, the presence of coenzyme A in mothers’ milk that exhibits TH2 adjuvant activity is correlated with a higher prevalence of atopic dermatitis (AD) in children^22^. The “atopic” trait mounts a TH2 response to common ubiquitous allergens^23,24^. Although adult European AD is characterized by a predominant expression of Th2 cytokines, pediatric and Asian-origin AD patients have a mixture phenotype comprised of Th2 and Th17 cytokine responses^25^.

While the pathogenesis of AD has been mostly linked to an altered adaptive immune response, the role and involvement of the innate immune system have not been widely examined^26–28^. This innate immune system is programed to recognize series of molecular patterns: (i) pathogen‐associated molecular patterns (PAMPs), and (ii) damage‐associated molecular patterns (DAMPs)^29–32^.

Innate lymphoid cells (ILCs) are lymphocytes that have lymphoid morphology but lack an antigen receptor^33,34^. Instead of antigen stimulation, ILCs promptly respond to various cell-derived factors, such as cytokines, which are produced by other cells in response to PAMPs and DAMPs^35,36^. Besides providing an early immune protection against pathogen infections, ILCs play a crucial role in inflammation, tissue repair and remodeling in multiple anatomical compartments, particularly at mucosal surfaces^37,38^. ILCs are currently categorized into three distinct populations based on their cytokine secretion profile and expression of transcription factors. Group 1 ILCs (ILC1) express T-bet and produce interferon-γ-mediated responses against pathogens, while Gata3^+^Group 2 ILCs (ILC2s) are involved in parasite expulsion, allergenic responses and, in skin, through the production of type 2 cytokines, IL5 and IL13, progress atopic dermatitis^39^.

Group 3 ILCs (ILC3s) are defined by the expression of the transcription factor RORγt and contribute to the maintenance and repair of epithelial tissues through IL22 upregulation^40^. In skin, ILC3s are associated with the pathogenesis of psoriasis through IL23 stimulated IL17 and IL22 production^41,42^. ILC3s are further classified into several subsets with distinct but overlapping phenotypic and functional markers. Among the few subsets, lymphoid tissue inducer (LTi) cells promote the development of lymph nodes and Peyer’s patches during embryogenesis. Intestinal LTi cells do not generally express lineage-specific markers but can express the T cell surface molecule CD4 and MHC class II. Two other subsets of group 3 ILCs are defined by a presence or absence in the expression of natural cytotoxicity receptors, NCRs, including NKp46 and NKp44^43,44^. It is now evident that ILCs are pivotal actors in allergic and inflammatory diseases pertaining to various organs including the intestine and skin. Despite the similarity of skin and intestine as barrier organs, the effect or a link between these two tissue-resident ILCs to one another has not been elucidated.

In the current study, we assessed the effect of breastfeeding in relation to atopic dermatitis in children. We detected DAMPs activity in breast milk of mother cohort of which children developed AD/eczema. We, however, could describe statistically “no evidence” on the association between DAMPs activity and AD in children. However, this might be a first case to detect DAMPs activity in breast milk, then, we wonder what the nature of DAMPs molecule is. By non-targeted metabolomic analysis, DAMPs(+)breast milk particularly contained high amount of long-chain saturated fatty acids (LCSFA) compared to healthy group breast milk. We demonstrated that LCSFA are potent DAMPs that initiate inflammatory events in vitro and in vivo. To elucidate the molecular pathogenesis, we created the atopic dermatitis mice model and monitored eczema in breastfed offspring. We demonstrated that mice breastfed with milk high in LCSFA develop higher occurrence of skin eczema, high TEWL, high IgE/mast cell and showed profound changes in the intestinal innate lymphoid cells type3 (ILC3) which travel from gut to skin.

## Results

### Mother’s milk possesses DAMPs activity containing high concentrations of long-chain saturated fatty acid

**Figure 1:**
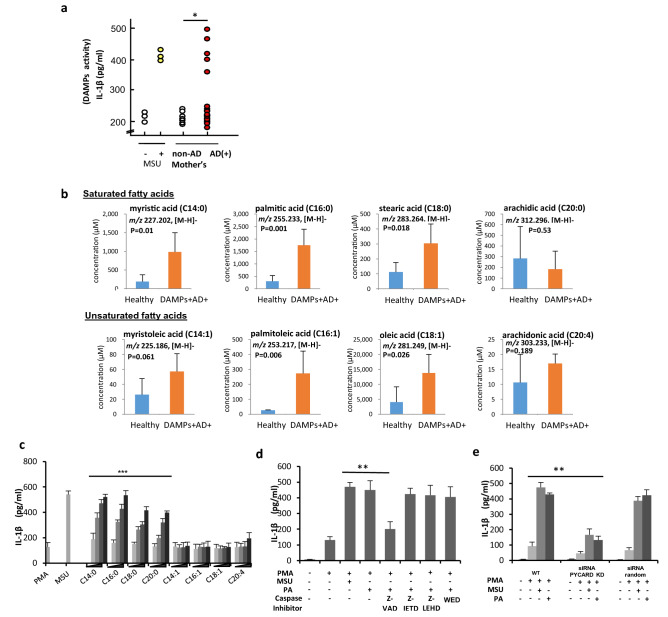
Breastmilk containing high amounts of long-chain saturated fatty acids is associated with atopic dermatitis. (**a**) DAMPs assay. PMA-differentiated THP-1 macrophages were treated with mother’s milk of non-AD or AD(+) infants (n = 75) for 12 h. Monosodium Urate, MSU was used as positive control. IL-1β production in cell culture supernatant was determined by ELISA. (**b**) Quantity of long-chain saturated and unsaturated fatty acids (C14 to C20) in healthy or DAMPs+ AD+ milk was determined by targeted metabolomic analysis (C8 separated). DAMPs+ AD+ milks are samples of AD+ infants and of which their respective mother’s milks are also positive for DAMPs assay. (**c**) DAMPs assay was performed with a series of long-chain saturated (C14:0, myristic acid, C16:0, palmitic acid, C18:0, stearic acid, C20:0, arachidic acid) and unsaturated (C14:1, myristoleic acid, C16:1 palmitoleic acid, C18:1, oleic acid, C20:4, arachidonic acid) fatty acids at an increasing concentration at 50, 100, 150 and 200 μM. (**d**) DAMPs assay of palmitic acid (PA) treated THP-1 macrophages in the presence of caspase inhibitors Z-VAD, Z-IETD, Z-LEHD and wedelolactone, WED. e, DAMPs assay using wild-type, pycard-Asc knockdown or random siRNA knockdown THP-1 macrophages. Results shown are representatives of at least three independent experiments. Data are represented as mean ± SD. *P < 0.05, **P < 0.01, one-way ANOVA and Student-t test (only in **a**). Data are analyzed with Prism8 (version 8.4.3, https://www.graphpad.com/scientific-software/prism**).**

In the current study, we assessed the effect of breast milk on the innate immune response and AD in children. A first cohort of 900 newborn babies was monitored for AD, and milk samples from which breastfed infants developed AD after 6 months were labelled as AD(+)^44–47^. 75 AD (+) and 75 AD(−) subjects were randomly selected and tested for the presence of damage-associated molecular patterns (DAMPs). Approximately 7% (5/75) of AD(+) milk collected as first milk (colostrum) after birth showed the ability to induce the production and release of the inflammatory cytokine interleukin-1β(IL-1β), which is similar to or stronger than a known DAMPs, the crystal form of monosodium uric acid (MSU), when used to treat phorbol myristate acetate-differentiated THP-1 macrophages (Fig. 1a). As a second cohort study, breast milk from individuals (n = 268) were further examined at three sampling points: colostrum, 1 month and 6 months. We found that the DAMPs activity in the milk samples pertaining to an individual are highly varied. Some mother’s milk has no DAMPs activity for all three points during the 6 months’ lactation period, while some possess an ability to induce IL-1β production at least in one or combinations of the three sampling time points (Supplementary Fig. S1a,b). As the presence of DAMPs in mother’s milk was highly varied among individuals within the 6-months lactation period, statistically, it is difficult to determine the correlation between AD and DAMPs activity in breast milk. Some AD(-) breast milk also indicates as DAMPs(+). However, this might be a first case to detect DAMPs activity in breast milk, therefore, we wonder what a nature of this DAMPs molecule(s) is.

We first examined the possibility of the presence of several known DAMPs. HMGB1 protein, ATP and MSU in mother’s milk samples were depleted by boiling or trichloroacetic acid (TCA) precipitation, apyrase and uricase pre-treatment, respectively before being used for DAMPs assay. Depletion of these DAMPs still elicited IL-1β production indicated presence of other non-protein DAMPs as a causative factor in the AD(+) milk (Supplementary Fig. S1c). Therefore, we performed a global metabolite analysis (non-targeted metabolomics mass spectrometry analysis). Although the PCA-score plot showed that metabolomic variations between the DAMPs(+)AD(+) and healthy samples were very small, the orthogonal partial least square (OPLS) analyses succeeded in separating the characteristic profile of each group, suggesting that small differences existed between groups (Supplementary Fig. S2, S3 and Supplementary Table 1, analysis of 1st cohort sample). By using these spectra, differences were revealed among fatty acids between milk from the DAMPs(+)AD(+) and the healthy group (Supplementary Fig. S4, S5, S6). The quantity of long-chain fatty acids in the DAMPs(+)AD(+) group was at least two-fold higher (Fig. 1b) than that in the healthy group, suggesting that fatty acids were responsible for the positive DAMPs activity in AD(+) milk. We also performed in vitro analysis to examine a series of fatty acids with different carbon chain lengths (C14–C20) and found that, principally, long-chain saturated fatty acids (LCSFA) are DAMPs capable of inducing IL-1β production (Fig. 1c) by activating NLRP3-PYCARD and caspase 1 (Fig. 1d,e). This result is in consensus with previous reports that palmitic acid can promote IL-1β production through NLRP3^48,49^. LCSFA-induced IL-1β production was inhibited by the presence of the mono-unsaturated fatty acids (USFA), irrespective of their carbon chain length (Supplementary Fig. S7). Hence, we conclude that LCSFAs are potent DAMPs when present at high concentrations and are ameliorated by the presence of USFA.

### HR-1 mice model of breastmilk feeding containing high LCSFA reenact the occurrence of eczema in breastfed offspring

**Figure 2:**
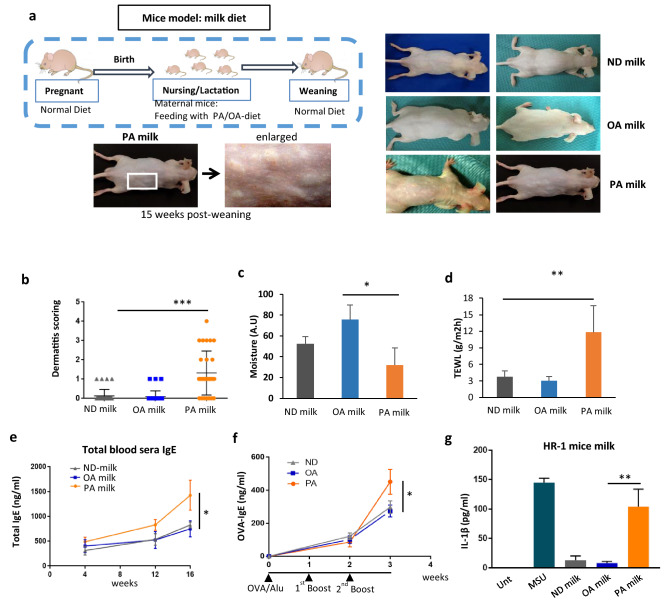
HR-1 mice offspring fed with maternal milk high in palmitic acid developed eczema. (**a**) Mice model of milk feeding. At 19 days nearing labor, pregnant HR-1 mice were fed with normal diet (ND) or chow diet added with 8% (w/w) saturated fatty acid, palmitic acid (PA) or 8% (w/w) unsaturated fatty acid, oleic acid (OA). In the case of PA milk or OA milk group, after birth, offspring from ND maternal mice were swapped to be breastfed by mice given diet high in PA or OA. Food was changed to normal diet when offspring started to feed on their own. (**b**) Dermatitis score of mice (n = 32 mice/group) at 15 weeks post weaning (p.w.) were evaluated base on (1) erythema/ hemorrhage, (2) scarring/dryness, (3) edema, (4) excoriation/erosion, scored as 0 (none), 1 (mild), 2 (moderate) and 3 (severe). The sum of the individual scores was taken as the dermatitis score. (**c**) Skin moisture and (**d**) transepidermal water loss of mice (n = 5 mice/group) at 15 weeks p.w. were determined using the Delfin MoistureMeterSC and Delfin VapoMeter, respectively. TEWL measurements were made on the lesional area of the skin. (**e**) Total blood serum IgE (n = 5 mice/group) was determined using the IgE FlowCytomix Simplex kit (eBioscience) according to the manufacturer’s manual. (**f**) Mice at 4 weeks p.w. (timeline = 0 week) (n = 5 mice/group) were sensitized with an intraperitoneal (i.p.) injection of 10 μg of OVA and 1 mg of alum, and re-sensitized twice at one week intervals. OVA-specific IgE in blood serum was determined with DS mouse IgE ELISA (OVA). (**g**) Milk of maternal mice given OA or PA diet (n = 3) were collected and examined for DAMPs activity with IL-1β ELISA. Results shown are representatives of at least three independent experiments. Data are represented as mean ± SD. *P < 0.05; **P < 0.01; ***P < 0.001, one-way ANOVA. Data are analyzed with Prism8 (version 8.4.3, https://www.graphpad.com/scientific-software/prism).

Based on the above-mentioned results, we wonder whether early influences of DAMPs (+) breast milk affect the pathogenesis of immune disorders, including atopic allergies in later life. We pursuit the possibility as DAMPs (+) breast milk is one of the factors of multifactorial AD. Therefore, we designed a mouse model utilizing HR-1 hairless mice with normal immune systems to evaluate the incidence of AD in relation to a breast milk diet (Fig. 2a). HR-1 mice lose their hairs from the back of their heads to tails at 2 to 3 weeks old which enables for easy observation on skin condition^50,51^. In the normal diet (ND) group, pregnant mice were fed a chow diet. Mice in the LCSFA group were fed a diet rich in LCSFA as they neared labour, containing 8% (w/w) palmitic acid (PA) or oleic acid (OA) as a control of long-chain mono-unsaturated fatty acid (LC-MUFA) (Supplementary Table 2). After birth, offspring from the ND mice were breastfed by maternal mice fed with diet high in PA (PA milk) or OA (OA milk) and returned to ND when the offspring were weaned (baby-swapping experiment). At 15 weeks post-weaning, offspring consuming the PA milk showed an increased incidence of skin complications, such as rashes or scratch wounds with bleeding (Fig. 2a). These skin complications were not observed in the normal milk diet or OA milk diet group. PA milk mice showed higher dermatitis scoring (Fig. 2b), lower skin moisture content (Fig. 2c), and higher trans-epidermal water loss (Fig. 2d). Consistent with the hallmark of AD, increased blood sera IgE (Fig. 2e and Supplementary Fig. S9) and antigen OVA-specific IgE (Fig. 2f) levels in high PA milk mice were observed by three weeks post-weaning. It is better to mention that, with this experimental system, it takes 15 weeks of post-weaning to develop skin complication, however by using adult mice with direct supplementation of PA diet, it takes at least 6 months to a year to develop similar skin symptoms.

Similar to that seen in the human cohort study, breast milk of mice fed a diet rich in PA also showed high DAMPs activities when introduced to human THP-1 macrophages (Fig. 2g). Certainly, the quantity of palmitic acids in lactating mice given diet high in palmitic acid was few folds higher than that of oleic acid or normal diet mice (Supplementary Fig. S8d). In addition to PA, we examined other LCFSA, including myristic acid and stearic acid, to determine their relationship with the occurrence of skin eczema in breastfed offspring (Supplementary Fig. S8a, S8c). Although the frequency of offspring developing eczema was not as high as that in offspring consuming PA milk, other LCSFA may induce a similar incidence of skin complications at an average of 15 weeks post-weaning. Taken together, the HR-1 mouse model of breastfeeding showed a risk of AD and other skin complications in offspring consuming milk high in LCSFA. We also constructed HR-1 mice with Rag1 knockout, referred to as *Rag-1*^−*/*−^ HR-1. These *Rag1*^−*/*−^ mice lacking an acquired immune system also developed similar skin complications when administered the PA milk diet (Supplementary Fig. S8b, S8c), suggesting that innate immune cells play an important role and are involved primarily in this PA milk induced AD mouse model.

### Early exposure to LCSFA promote Intestinal inflammation via type 3 innate lymphoid cells

**Figure 3:**
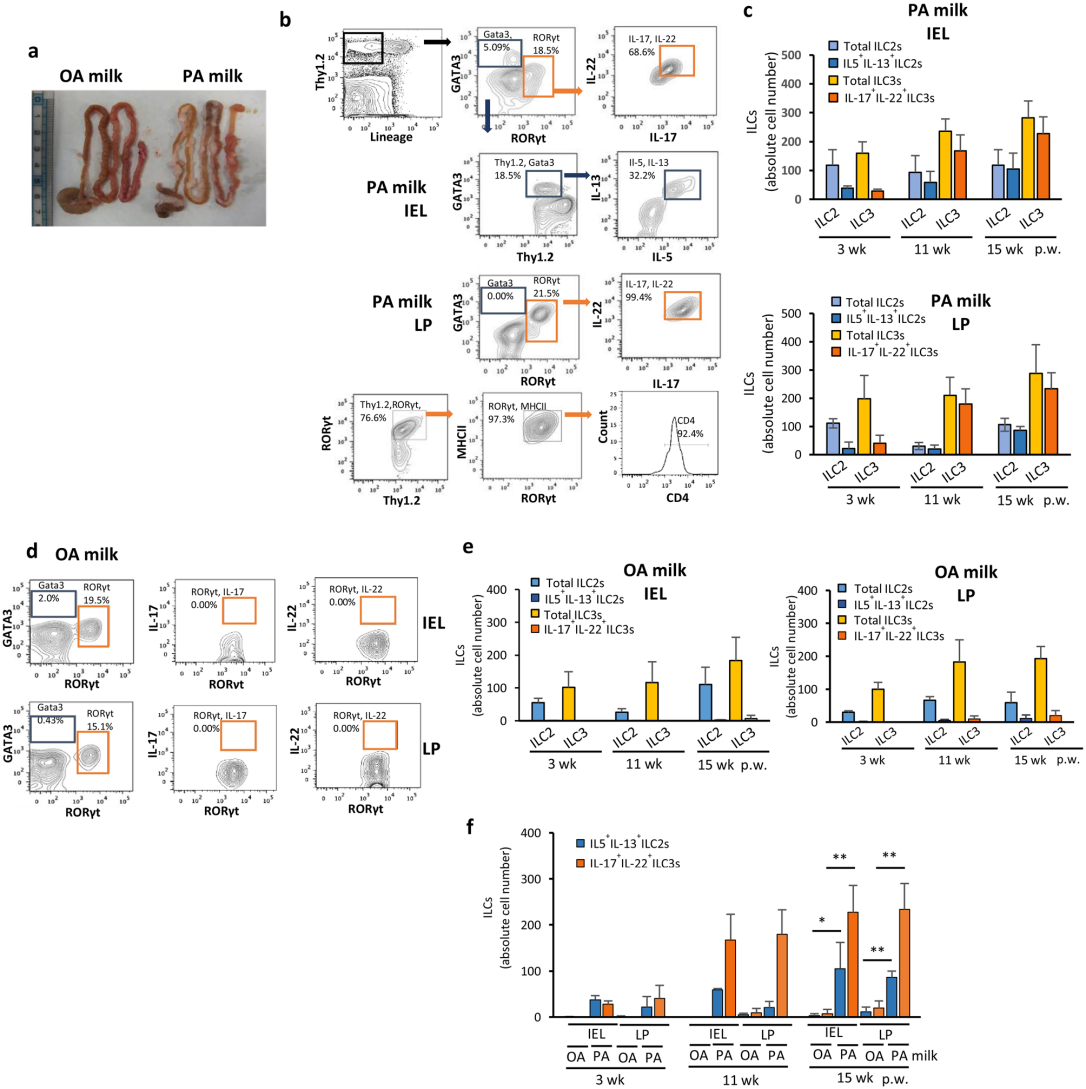
PA milk diet induced inflammatory ILCs in the small intestine. (**a**) Pictures of the entire intestines of PA milk and OA milk mice taken at 15 weeks post weaning (p.w.). (**b**,**d**) Representative FACS analysis of (**b**) PA milk and (**d**) OA milk mice at 15 weeks p.w. Small intestine intraepithelial layer (IEL) and lamina propria (LP) of mice at 3, 11 and 15 weeks p.w. were isolated via percol gradient centrifugation. Cells were stained with Thy1.2 and lineage markers (NK1.1, T cell receptor TCRβ and TCRγδ, CD11b, CD19, CD8, Gr-1 and Ter119). ILCs were gated by Lin^−^ and Thy1.2^+^ expression, intracellularly stained for GATA3 (ILC2s) and RORγt (ILC3s), and interleukins (IL-5, IL-13, IL-17A and IL-22). (**c**,**e**) Absolute ILCs number and their respective interleukins in the IEL and LP at the indicated p.w. (n = 5 mice/group). (**f**) Comparison of the inflammatory ILCs in the IEL and LP of OA and PA milk. Results shown are representatives of at least three independent experiments. Normal diet group (ND) data is almost same as OA milk group results. Data are represented as mean ± SD. *P < 0.05, **P < 0.01, one-way ANOVA. Data are analyzed with Prism8 (version 8.4.3, https://www.graphpad.com/scientific-software/prism), and FlowJo (version 10.7.1, https://www.flowjo.com/solutions/flowjo/downloads).

Next, to determine the molecular mechanisms underlying the PA milk induced AD, we first investigated the changes in intestinal innate immunity. Innate immunity is of primary importance during early life development, when interactions with the environment occur at the mucosal surfaces^52,53^. Innate lymphoid cells (ILCs) have been increasingly recognized as important participants in immune homeostasis, inflammation, and the pathogenesis of inflammatory, allergic, and autoimmune disorders^54,55^. Thus, we examined innate immune cells from small intestine intraepithelial cells (IELs) and the lamina propria (LP). Small intestines collected from PA milk offspring mice with severe eczema appeared enlarged and longer, with an increased mass of mesenteric fats compared to that from OA milk offspring mice (Fig. 3a). ILC populations were assessed by flow cytometry and stained for lineage-negative and Thy 1.2-positive cells. Cells showing high expression of Gata3 and RORγt were gated as ILC2 and ILC3, respectively. Although T-bet-positive ILC1 cells were expressed in both IEL and LP, there were no significant differences between the three groups of normal milk, OA milk, and PA milk offspring mice.

Representative flow cytometry figures for the analysis of intestinal IELs and LP of offspring mice at 15 weeks post-weaning are shown. We found that compared to OA milk mice (Fig. 3d,e), ILC3s were increased in both the IEL and LP of PA milk mice at 11 and 15 weeks after weaning (Fig. 3b,c). At 11 weeks post-weaning, approximately 50% of the ILC3s in both IEL and LP were double producer for the inflammatory cytokines IL-17A and IL-22. At 15 weeks post-weaning, all ILC3s from the LP of PA milk mice were IL-17A^+^ and IL-22^+^, suggesting increased inflammation over time, which was attributed by the ingestion of milk with high levels of PA, but not OA (Fig. 3f). In our experimental HR-1 mouse model, ILC2s accounted for a small percentage of cells in the gut IEL or LP in early life. However, in PA milk mice of 15 weeks post-weaning, nearly all ILC2s were double positive for IL-5 and IL-13 (Fig. 3b,c). These data suggest that milk with high levels of LCSFA promote pro-inflammatory ILC responses in the intestinal microenvironment. Results are in agreement with several previous reports that, at 15 weeks, about 20% of ILC2 was detected among total gut ILCs. Percentages of the ILCs and their respective interleukins in the IEL and LP of Normal diet milk samples were almost same as that of OA milk results.

### Skin inflammation relates to an increase in IL-17A and IL-22 producing ILC3s in the dermis

**Figure 4:**
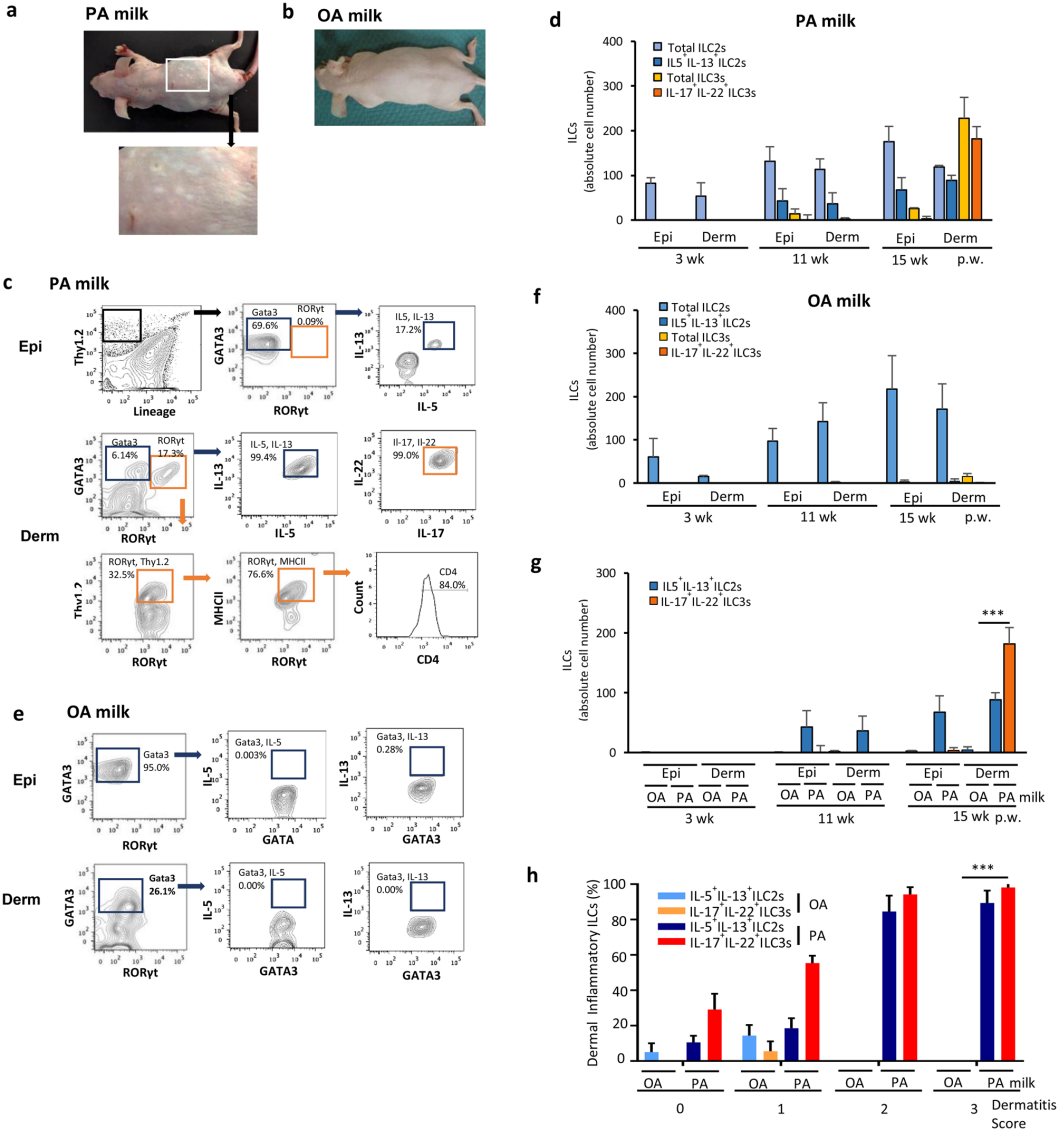
Inflammatory ILC3s was increased in the dermis of PA milk mice. (**a**,**b**) Pictures of (**a**) PA milk and (**b**) OA milk mice taken at 15 weeks post weaning (p.w.). (**c**,**e**) Representative FACS analysis of (**c**) PA milk and (**e**) OA milk at 15 weeks p.w. Epidermis and dermis ILCs of mice at 3, 11 and 15 weeks p.w. were isolated separately. Cells were stained with Thy1.2 and lineage markers (NK1.1, T cell receptor TCRβ and TCRγδ, CD11b, CD19, CD8, Gr-1 and Ter119). ILCs were gated by Lin^−^ and Thy1.2^+^ expressions, intracellularly stained for GATA3 (ILC2s) and RORγt (ILC3s), and interleukins (IL-5, IL-13, IL-17A and IL-22). (**d**,**f**) Absolute ILCs number and their respective interleukins in the IEL and LP at the indicated p.w. (n = 5 mice/group) were represented in bar graph as means ± SD. (**g**) Comparison of the inflammatory ILCs in the epidermis and dermis of OA and PA milk. In OA-milk data, most of ILC cell number is not detected. (**h**) Percentages of inflammatory ILC2s and ILC3s in association with dermatitis score of OA or PA milk mice at 15 weeks post-weaning. Results shown are representatives of at least three independent experiments. Normal diet group (ND) data is almost same as OA milk group results. Data are represented as mean ± SD. ***P < 0.001, one-way ANOVA. Data are analyzed with Prism8 (version 8.4.3, https://www.graphpad.com/scientific-software/prism), and FlowJo (version 10.7.1, https://www.flowjo.com/solutions/flowjo/downloads).

In parallel to the identification of ILC3 and ILC2 inflammatory cytokines in the gut of PA milk mice at 15 weeks post-weaning, these mice exhibited skin complications with pathogenesis similar to AD (Fig. 4a). Eczema was not observed in OA milk mice (Fig. 4b). The skin epidermis and dermis were isolated separately and examined for the innate lymphoid cell population by flow cytometry. Gata3-positive ILC2s appeared to be the major skin-resident ILCs in both the epidermis and dermis layers of HR-1 mice.

Offspring from the PA milk diet group that developed skin complications at 15 weeks post-weaning exhibited increased production of the ILC2 key cytokines IL-5 and IL-13 (Fig. 4c,d).

ILC2 cells population, however, isolated from offspring breastfed with milk of lactating mice fed a high OA diet, typically did not produce IL-5 or IL-13 cytokines (Fig. 4e,f). Our findings agree with those of previous reports regarding the involvement of ILC2 and type 2 cytokines in the pathogenesis of AD.

Interestingly, concomitant with the appearance of cytokine-producing ILC2s, ILC3s were detected in the dermis of PA milk mice (Fig. 4c,d), which were not present neither in the group of OA milk mice nor at 11 weeks (pre-symptomatic stage) of PA milk mice (Fig. 4c–g). We found that the severity of the skin symptoms corresponded to the appearance and percentage of inflammatory cytokines producing ILC2s and ILC3s (Fig. 4h).

For instance, nearly 100% of the total ILCs were double positive for inflammatory cytokine production when the skin condition was severe with scratches and bleeding (dermatitis score more than 2, 3), but in mice with milder skin conditions, ILC-derived cytokines accounted for less of the total population. Similarly, in the gut, mice with severe skin complications showed a higher incidence of intestinal IL-22^+^/IL-17^+^ ILC3 cells. These results suggest that mice offspring breastfed with milk high in SFAs resulted in increased inflammation in the intestine and skin. Importantly, we found that the appearance and increase of IL-17A^+^/IL-22^+^ ILC3s always began in the small intestine at 11 weeks post-weaning (pre-symptomatic stage) prior to appearance in the skin at 15 weeks post-weaning. This suggests that initiation of inflammation occurs in the intestinal microenvironment by LCSFA either directly or indirectly, which may lead to, or trigger, a chain reaction that induces AD.

### HR-1 mice offspring breastfed with milk high in PA showed intestinal and skin histopathology resembling atopic dermatitis

**Figure 5:**
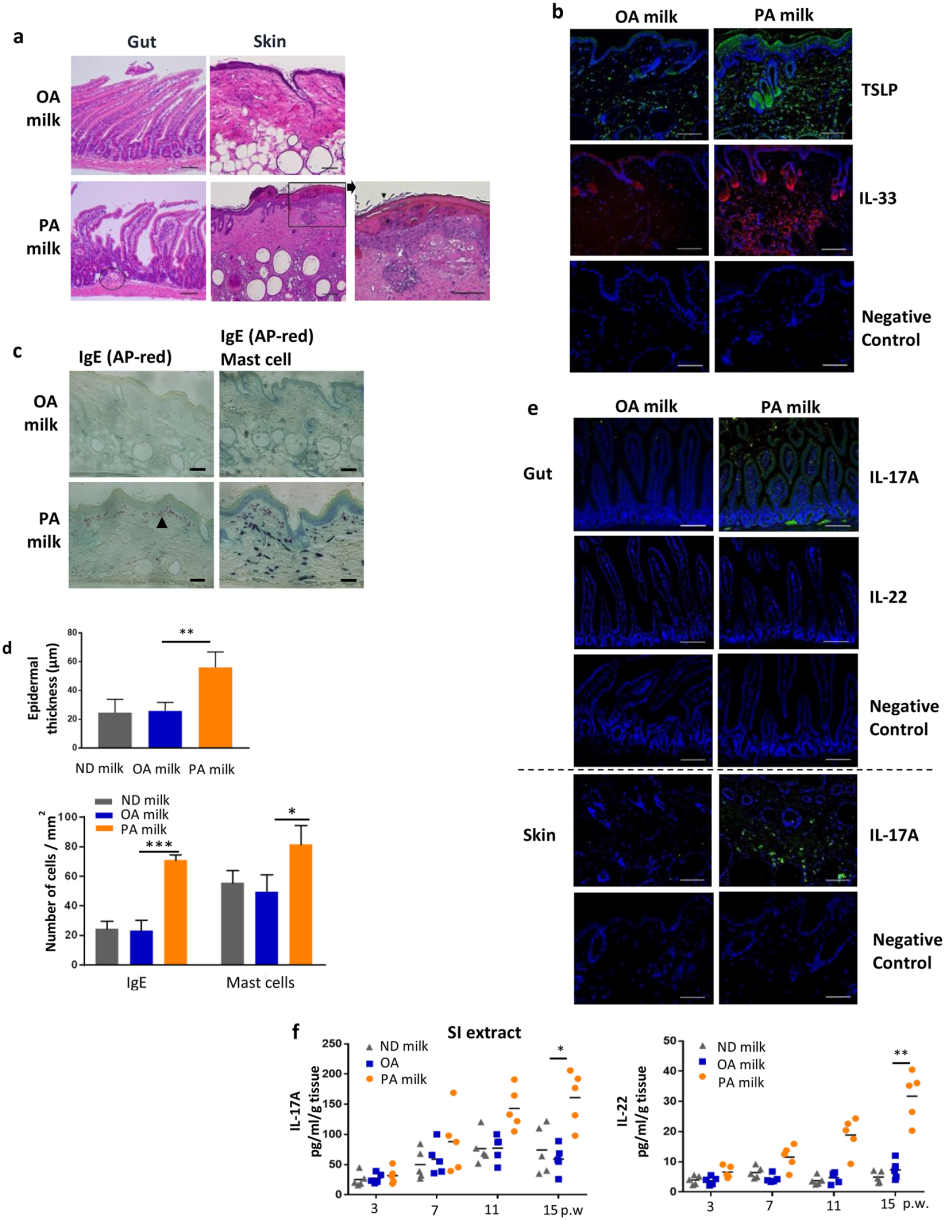
PA milk diet induced inflammation in the gut and skin. (**a**) Hematoxylin and eosin staining of the small intestine and skin of OA milk or PA milk mice at 15 weeks post-weaning (p.w.). Gut of PA milk mice was observed with mucosal or submucosal inflammation, accompanied with inflammatory cells infiltration (black circle). A section of the PA milk skin is enlarged showing histopathology of atopic dermatitis. Black and white arrows indicate parakerotosis and spongiosis, respectively. Asterisk indicate neutrophil containing pustule. Scale bars, 100 µm. (**b**) Representative immunofluorescence staining for TSLP and IL-33 in skin of mice at 15 weeks p.w. Scale bars, 100 µm. (**c**) Representative immunohistochemical staining of IgE (fast red chromogen) and mast cells (toluidine blue) in the skin of mice at 15 weeks p.w. Scale bars, 100 µm. (**d**) Epidermal thickness and number of IgE-stained or mast cells were calculated from 10 randomly selected areas and shown as the mean number of cells per millimeter squared ± SD (n = 3 mice/group). (**e**) Representative immunohistochemical staining of IL-17A and IL-22 (DAB chromogen and hematoxylin) in small intestine and skin of mice at 15 weeks p.w. Scale bars, 100 µm. (**f**) ELISA quantitation of IL-17 and IL-22 of small intestine (SI) tissue extracts at indicated p.w. (n = 5 mice/group). *P < 0.05, **P < 0.01, ***P < 0.001, one-way ANOVA. Data are analyzed with Prism8 (version 8.4.3, https://www.graphpad.com/scientific-software/prism), and ImageJ (version 1.53, https://imagej.nih.gov/ij/index.html).

Next, we examined small intestine and skin histopathology. The small ileum of PA milk mice showed inflammatory cell infiltration in the mucosal and submucosal layers. In addition to the increased thickness of the epidermal layer, histological features of AD, such as parakeratosis, dyskeratosis, and spongiosis, were observed in PA milk mice, but not in OA or normal milk offspring mice (Fig. 5a). Furthermore, an evident increase was observed for the skin thymic stromal lymphopoietin (TSLP) and IL-33, cytokines that are highly associated with the pathogenesis of AD (Fig. 5b, Supplementary Fig. S12a). Immunostaining revealed a marked increase in IgE complex and mast cells that mostly colocalized at the dermis of PA milk mice (Fig. 5c,d). We found that mice with very severe eczema accumulated these IgE complexes, which were lined up just beneath the epidermal stratum basal layer in the papillary layer of the dermis. Mice with milder eczema seemed to have IgE complexes dispersed within the reticular layer of the dermis. This suggest that an increase in the allergy-mediator IgE immune complex and a movement from the dermis nearing the epidermis may play an important role in the pathogenesis and severity of atopic dermatitis that reflects upon the surface of the epidermis. In agreement with the flow cytometry data, immunostaining and quantitation of the small intestinal extracts from PA-milk mice showed a significant increase in the inflammatory cytokines IL-17A and IL-22 (Fig. 5e,f, Supplementary Fig S12b).

**Figure 6:**
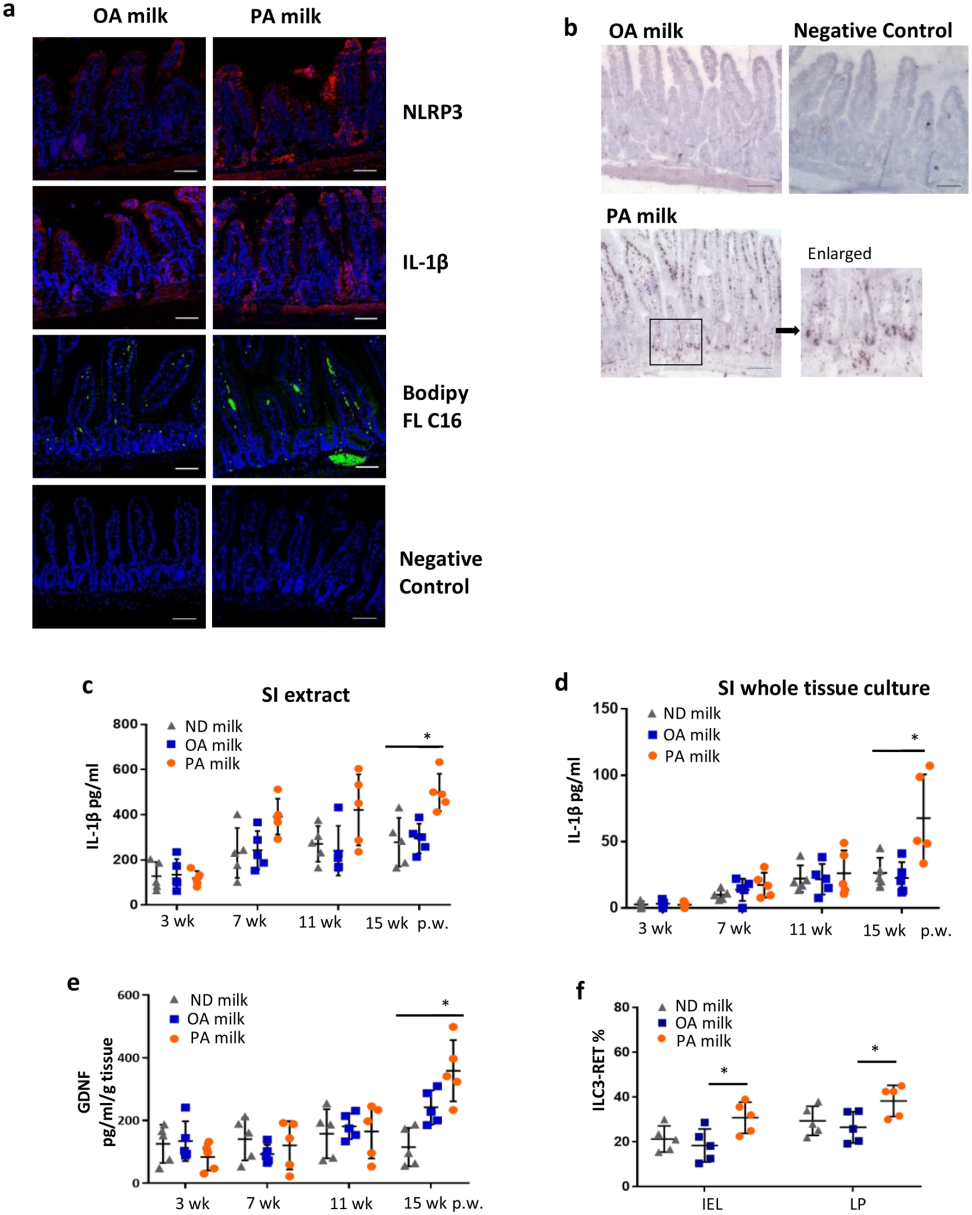
PA-milk diet induced inflammation in the gut and skin. (**a**) Representative immunofluorescence staining for NLRP3 (red), IL-1β (red) and Bodipy FL C16 (green) in the small intestine of mice at 15 weeks post-weaning (p.w.). Nuclei were stained with DAPI (blue). Scale bars, 100 µm. (**b**) Oil red O lipid staining of the small intestines of mice at 15 p.w. ELISA quantitation of IL-1β (pg/ml) in (**c**) small intestine (SI) extracts and (**d**) whole tissue culture taken from mice at indicated p.w. (n = 5 mice/group). SI extracts were obtained by lysis treatment and sonication. For whole tissue culture, small intestine was cut into small pieces and cultured on 24-well tissue dish for 18 h before the collection of supernatant for IL-1β quantitation. (**e**) ELISA quantitation of GDNF in small intestine extracts at indicated p.w. (**f**) Percentages of ILC3-RET expression in IEL and LP of mice at 15 weeks p.w. (n = 8 mice/group). *P < 0.05, **P < 0.01, one-way ANOVA. Data are analyzed with Prism8 (version 8.4.3, https://www.graphpad.com/scientific-software/prism), and ImageJ (version 1.53, https://imagej.nih.gov/ij/index.html).

Similarly, the inflammasome NLRP3 and its secretory cytokine product IL-1β were evidently increased by two-folds in PA milk mice (Fig. 6a,c, Supplementary Fig. S12c). Interestingly, when the small intestines were harvested, cultured in medium, and then evaluated for IL-1β production, we found that the small intestines of PA milk mice produced larger amounts of IL-1β in the overnight medium (Fig. 6d). A significant difference was observed in PA milk at 15 weeks post-weaning in the mice, suggesting that the small intestines exposed to PA were vulnerable and had an inflammatory-inductive tendency, which persisted until adulthood. Moreover, several inflammatory cytokines and chemokines were also increased in the blood sera of PA milk mice (Supplementary Fig. S10, S11). Oil-red O staining and BODIPY C16 immunofluorescence staining indicated high lipid accumulation in the small intestine of PA milk mice (Fig. 6a,b, Supplementary Fig. S12c, S12d). Accumulation of the C16-lipid in the basal and ileum of PA milk mice was mostly accompanied by inflammatory cell infiltration, suggesting that the presence of SFAs induces a local inflammatory response in situ.

### Pro-inflammatory ILC3s production in small intestine of PA-milk is associated with the IL-1β-GDNF stimulation of ILC3 RET

Glial cell-derived neuroregulators control ILC3 in the gut and are critical for regulating mucosal homeostasis^56,57^. We found that the glial-derived neuroregulator, GDNF, was increased in small intestine extracts from PA milk mice (Fig. 6e). Concomitantly, an increase in the neuroregulatory receptor, RET, was observed in the ILC3 of PA milk mice LP (Fig. 6f). Under homeostatic conditions, ILC3s respond to local cues to maintain tissue homeostasis and restrict inflammatory responses^58,59^. Thus, the LCSFA PA may directly stimulates intestinal cells, resulting in an inflammatory response via NLRP3 activation. As a result, IL-1β triggers GDNF production, leading to higher RET expression in ILC3s. In agreement with a previous report, we believe PA-induced IL-1β production is partly involved the enteric glial-ILC3 axis to regulate and maintain intestinal homeostasis upon inflammation. However, it would also be interesting to examine other possible mechanisms contributing to increased inflammatory ILC3s.

### Trafficking of inflammatory ILC3s from the intestine to the skin increases incidence of atopic dermatitis

**Figure 7:**
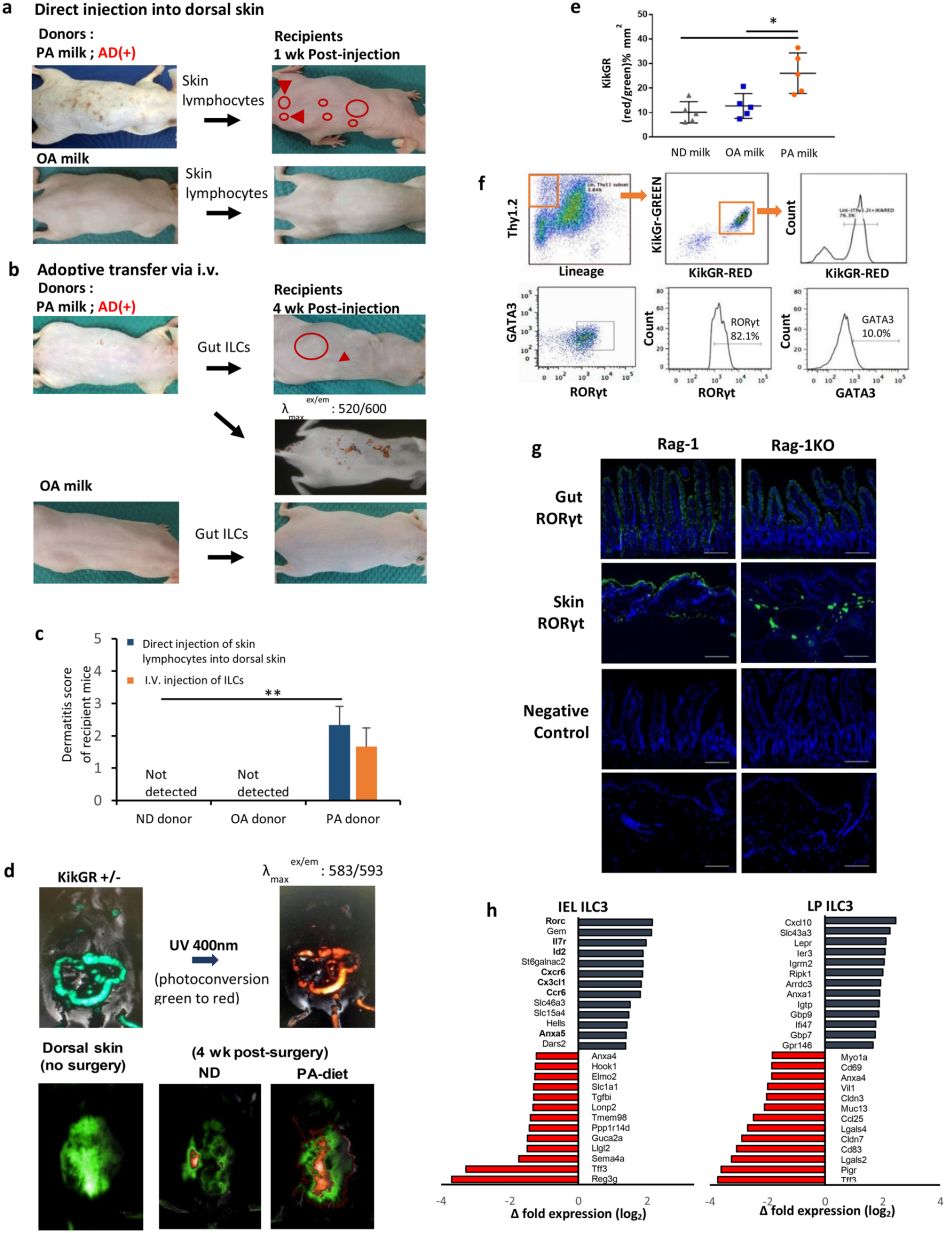
Trafficking and migration of pro-inflammatory ILC3s from small intestine to skin potentiates incidence of AD in PA-milk mice. (**a**) Skin lymphocytes were isolated from PA milk mice with eczema or OA milk mice, and directly injected into the dorsal skin of healthy recipient mice. (**b**) Gut ILCs were sorted from IEL and LP of PA milk or OA milk mice, labelled with Qtracker (Red) and performed adoptive transfer by intravenous injection into healthy recipient mice. (**c**) KikGR transgenic mice were subjected to surgery, where the small intestine was taken out and treated with UV at 400 nm (USIO SP-11). The presence of the red-photoconverted intestinal lymphocytes on the dorsal skin were detected by fluorescence microscopy (NightOwl, Berthold technologies). **P < 0.01, one-way ANOVA. (**d**) The ratio of red to the green fluorescence areas (millimeter per squared) on the dorsal skin (n = 5 mice/group) was calculated using the IndiGO software. (*P < 0.05). (**e**) Dorsal skin (epidermis) of KikGR post-surgery mice with red-photoconverted cells were subjected to cell sorting for RED^+^ cells. Isolated Lin^−^Thy1.2^+^KikGR-RED^+^ cells were then intracellularly stained for RORγt. Representative FACS analysis of isolated skin lymphocytes, gated for ILC3s (RORγt), KikGR green and red fluorescence (n = 6 mice/group). *P < 0.05, **P < 0.01, one-way ANOVA. (**f**) Representative of immunofluorescence staining for RORγt (green) in the gut and skin of PA milk and Rag1 KO, PA milk mice. Nuclei were stained with DAPI (blue). Scale bars, 100 µm. (**g**) Bar graph indicates a list of selected genes with significantly up-regulated expression (red) or down-regulated expression from ILC3s isolated from intraepithelial, IEL and lamina propria, LP of PA milk mice compared with ND mice. IEL and LP were isolated as described in the methods, and ILC3 were sorted with FACSAria III, gated for lineage negative, CD45^+^, Thy1.2^+^, CD25^+^ and Nrp1^+^ cells. Results shown are representatives of at least three independent experiments. Full list of RNA sequencing analysis can be found through the GEO accession number, GSE 124188. Data are analyzed with Prism8 (version 8.4.3, https://www.graphpad.com/scientific-software/prism), ImageJ (version 1.53, https://imagej.nih.gov/ij/index.html), FlowJo (version 10.7.1, https://www.flowjo.com/solutions/flowjo/downloads), and IndiGO (version 2.0.5.0, https://www.berthold.com/en/bioanalytic/products/in-vivo-imaging-systems/nightowl-lb983/).

As the appearance of the inflammatory ILC3s always appeared in the gut before the skin, we sought to examine if these intestine inflammatory ILCs may traffic from the gut to the skin. Directly transferred skin lymphocytes isolated from PA milk mice and injected into the dorsal skin of healthy recipient mice led to the development of skin symptoms, similar to eczema, at one-week post-injection, which was not observed in the recipients of normal or OA milk donor cells (Fig. 7a,c). At a later observation time, adoptive transfer of small intestine ILCs from PA milk via intravenous (i.v.) injection also resulted in the appearance of skin rashes and raised bumps at an average of five weeks post-injection (Fig. 7b,c). These donor-derived ILCs were incubated with Qtracker Red pre-injection, and the presence of these red cells on the skin surface was detected at one-week post-i.v.

To track the migration of gut lymphocytes, we utilized transgenic reporter mice expressing the photoconvertible fluorescent protein, Kikume Green–Red (KiKGR). KikGR is natively a green fluorophore that can be converted into a red fluorophore upon exposure to UV light^60–62^. KikGR mice were subjected to surgery, with the small intestines carefully removed and treated with UV light at 405 nm. Extra care was taken to protect the Payer’s patches and other body parts from UV exposure by aluminum foil shielding. Only surfaces of the small intestine exposed to UV treatment showed photoconversion from green to red. At one month post-surgery, intestinal-derived, photoconverted red cells were observed in the dorsal skin (Fig. 7d) and remained detectable for at least three months. This suggests that gut-derived innate lymphocytes reach and deposit at the skin layers. Interestingly, KikGr mice offspring fed a PA milk diet showed a higher percentage of intestinal lymphocyte migration to the skin compared to that of OA milk or ND mice (Fig. 7e). Further analysis of photoconverted cells isolated from the dorsal skin revealed that these intestinal derived cells of majority (82%) were RORγt^+^ ILC3s (Fig. 7f and Supplementary Fig. S13). This indicates that inflammatory innate lymphoid cells from the intestine have higher migration rates throughout the bloodstream and mobilize at the skin, which may be potential triggers or worsening factors of AD. The presence of ILC3s in both the small intestine and skin of Rag1^−/−^ HR-1 mice of PA milk was also observed by immunofluorescence staining against RORγt (Fig. 7g, Supplementary Fig. S12e) .

Finally, RNA sequencing analysis of ILC3s isolated from the small intestine IEL and LP layers of PA milk mice showed upregulation of transcription factors crucial for the development of ILC3s, including Rorc, Id2, and IL7r, which explains the tendency of ILC3s increase in PA milk mice compared with normal diet (ND). Additionally, the expression of several chemokine receptors, including CXCR6 and CCR6, was increased (Fig. 7h). Both these receptors and their ligands, CXCL16 and CCL20 are reported to be overexpressed in atopic dermatitis and lesional psoriatic skin, and contribute to the skin inflammation^63–66^. Overall, our data suggest that early exposure to a diet high in LCSFAs in infants promotes inflammation in the gut that extends to the skin layers, and over time when inflammation and inflammatory immune cells or cytokines accumulate, AD or other allergic response may occur.

### Gut microbiota of PA milk-fed mice is different from ND and OA milk-fed mice

**Figure 8:**
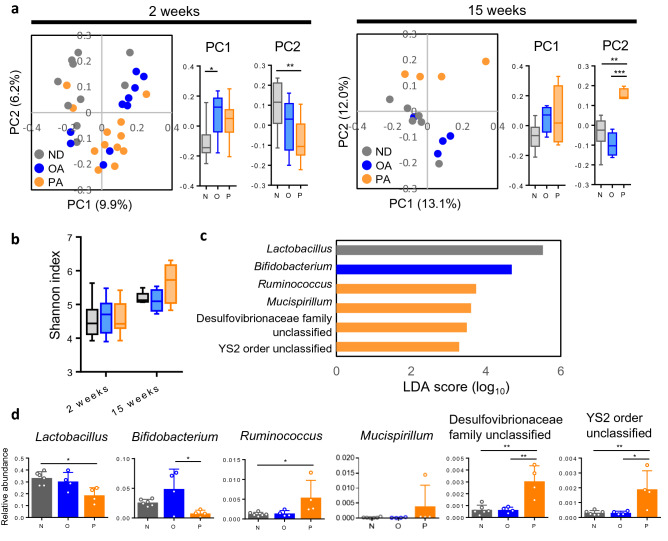
PA milk-fed mice have a unique microbiota different from ND and OA milk-fed mice. (**a**) PCoA of beta-diversity using the unweighted unifrac distance and (**b**) alpha diversity (Shannon index) of ND (*n* = 10), OA (*n* = 10) and PA (*n* = 12) milk-fed mice at 2 and 12 weeks of age. (**c**) Results of LEfSe analysis showing the genus that were significantly different in abundance among ND (*n* = 6), OA (*n* = 4) and PA (*n* = 4) milk-fed mice at 12 weeks of age. Gray, blue and orange indicate the bacteria with increased abundance in ND, OA and PA milk-fed mice, respectively. (**d**) Validation with one-way ANOVA analysis using relative abundance of the genus selected by LEfSe analysis. Data are represented as mean ± SD. *P < 0.05, **P < 0.01, ***P < 0.001, One-way ANOVA with post-hoc Tukey’s multiple comparison test. Data are analyzed with Prism8 (version 8.4.3, https://www.graphpad.com/scientific-software/prism) and QIIME2 (version 2017.6, http://qiime.org).

Dysbiosis of gut microbiota has been reported to associate with various disorders including allergic diseases including atopic dermatitis^67,68^. To investigate the relationship between AD development and gut microbiota in our HR-1 mice model of AD, we collected feces at 2 weeks and 12 weeks of age, before and after the appearance of skin lesion in PA milk-fed HR mice, from ND, PA and OA milk-fed mice. Beta-diversities (microbial diversity among the samples) in principal coordinate analysis (PCoA) at 2 weeks of age showed that the gut microbial diversity of ND milk-fed mice were significantly different from that of OA and PA milk-fed mice along PC1 and PC2 axis, respectively (Fig. 8a, left). Interestingly, at 12 weeks of age, gut microbial diversity of OA and ND milk-fed mice are closely plotted and significantly separated from that of PA milk-fed mice along PC2 axis (Fig. 8a, right). Alpha-diversities (microbial diversity within the sample), an indicator for dysbiosis, showed no differences among 3 groups (Fig. 8b), suggesting that PA milk-fed mice seemed not suffer from apparent dysbiosis. We next focused on gut microbiota of 12-week-old mice, after the skin lesion onset in PA milk-fed group, and compared the abundance of bacterial taxa in each group with a linear discriminant analysis effect size (LEfSe) analysis. We observed the increment of bacterial taxa belonging to genus *Lactobacillus* in ND milk-fed mice, genus *Bifidobacterium* in OA milk-fed mice, while genera *Ruminococcus* and *Mucispirillum*, and unclassified genera of Desulfovibrionaceae family and of YS2 order in PA milk-fed mice (Fig. 8c). Similarly to the analysis of beta-diversity at 12-week-old (Fig. 8a, right), the profile of bacterial genera in PA milk-fed mice is different from the other two groups (Fig. 8d); genera *Lactobacillus* and *Bifidobacterium* were significantly decreased, and the genera *Ruminococcus*, the unclassified genera of Desulfovibrionaceae family and the unclassified genera of YS2 order were significantly increased, in PA milk-fed mice.

## Discussion

Atopic dermatitis (AD) is a chronic, pruritic inflammatory skin disease, which generally develops in early childhood, affecting 10–20% of children in developed countries^69,70^. Epidemiological research on the relationship between diet and allergies has increased in the last decade but many uncertainties remain to be resolved. These include the role of breast-feeding, the role of different components of breast milk, the time in life when different dietary components exert their influence, and differences in sensitivity to dietary influences between individuals. These issues have been addressed in several papers and reviews^71,72^.

Lipids are one of the dietary components that seem to have an influence on the development of allergies^73,74^. For instance, diet of low n-3 polyunsaturated FA (PUFA) intakes, high proportion of n-6 PUFA, and high intakes of trans FA are related as risk factors for allergic diseases^21,75–78^. So far, most studies on the associations between lipid intakes and allergies conveys epidemiological significance whereas intervention studies are lacking. Our metabolomics data of breast milk revealed particularly high long-chain saturated fatty acids in the group of DAMPs(+) AD(+) milk/children. In fact, there are studies that indicated the presence of lipids in milk as ‘toxic’ or inflammatory milk^79,80^. And several studies revealed that palmitic acid can promote IL-1ß production through NLRP3^49,50^. By utilizing a mice model of breast milk feeding, we show that intake of high amounts of LCSFA (palmitic acid) in the first months of life may be critical for the development of AD or allergic diseases. It is conceivable that the ratio between LCSFA/LCUSFA is a key factor to develop skin inflammation/AD in our mice system (Fig. 1b and sFig S7). High fat diet has been mostly linked to the instigation of obesity and insulin resistance. Here, we propose a link that translate the inflammatory events induced by LCSFA via an involvement of the ILC3s.

Several reports have demonstrated that gut dysbiosis contributes to the pathogenesis of intestinal inflammation. Saturated fatty acids have been reported to cause gut dysbiosis^81^. Therefore, we analyzed the gut microbiota of ND, OA and PA milk-fed HR mice. Alpha-diversities of PA milk-fed mice were comparable to OA and ND milk-fed mice at 12 weeks of age when AD developed in PA milk-fed mice, suggesting no apparent dysbiosis in PA milk-fed mice; nevertheless, gut microbiota composition of ND and OA milk-fed mice were close to each other and different from that of PA milk-fed mice. These profiles were consistent with the AD onset phenotypes. PA milk-fed mice were characterized by a drastic decrease in the genus *Bifidobacterium* which is known as beneficial bacteria and contains some species which have suppressive effects of AD^82,83^, while some reports have shown that *Lactobacillus*, which was also slightly decreased in PA milk-fed mice, were comparable between AD and non-AD children^84^. By contrast, genus *Ruminococcus* was increased in PA milk-fed mice, consistent with a previous study reporting an increase in *Ruminococcus gnavus* in AD children^85^. Genus *Mucispirillum*, and unclassified genera of Desulfovibrionaceae family and YS2 order were also increased in PA milk-fed mice. There is no report showing the relationship of these bacteria with AD; their role in AD pathogenesis may not be significant given their very low abundance in the gut microbiota. Taken together, these results suggest that gut microbiota of AD-prone PA milk-fed mice is different from non-AD OA /ND milk-fed mice, and that a reduced *Bifidobacterium* may be associated with the development of AD.

ILCs are described with pivotal role in allergic, inflammatory and autoimmune diseases^86,87^. We observed that even several weeks post-weaning, offspring mice given milk high in palmitic acid remains with the tendency to build up inflammatory ILCs, which first appeared in the small intestine before the skin. An involvement of ILC2s in allergic diseases are commonly implicated^88–92^. However, a report described that spontaneous atopic dermatitis in mice with a defective skin barrier (Flg^ft/ft^ mice) is independent of ILC2 and mediated by IL-1ß^93^.

Our results show that besides ILC2s, the ILC3s responses and homeostasis are also altered. One of the reasons behind such changes involves the induction of GDNF and ILC3 RET expression by an increase of IL-1β, resulting in an increase of IL-22 production for homeostasis and maintenance of the gut. In our AD mice model, the gut ILC3s often increases before the ILC2s and the appearance of inflammatory ILC3s in the dermis of mice offspring that developed eczema seemingly correlated to the severity suggests that the type 3 innate immune response may be prerequisite to the AD pathogenesis. Although, we and others might be the first to report on a link between ILC3s and AD. Others reported, recently, that antibiotics-induced dysbiosis of gut microbiota play a crucial role in AD development through altered short-chain fatty acid production through Treg and ILC3^94^. In psoriasis, an increased proportions of ILC3s were observed in lesional skin and peripheral blood, and IL-22 is regarded as a key driver of epidermal thickening^95,96^. Also, another report indicate under homeostatic condition, ILC2 and ILC3 efficiently acquired palmitate^97^. This and our results suggest the connection between ILC2/3 and PA for the development of AD. It has been shown that increased ILC2s and ILC3s worsened atopic dermatitis^98^. However, we don’t have data that indicate increased PA act directly on ILCs.

We think that the increase of inflammatory ILC3s and the cytokines, IL-17 and IL-22 initiated in the gut may trigger the various inflammatory responses that progresses to atopic dermatitis. It is also interesting to elucidate as to whether the inflammatory ILC3s may initiate the activation of ILC2s, a cross-talk or plasticity between the two innate immune players.

As the ILC3s were not normally present in the skin, but were significantly increased with incidence of eczema, it is plausible that the inflammatory ILC3s in the dermis of PA milk mice were intestine derived ILC3s. Differences of circulating ILCs in the frequencies, phenotypes, and functions in the peripheral blood of allergic patients with rhinoconjunctivitis from nonallergic human subjects have been reported^99^. We think that PA milk induced inflammation may lead to a difference in the dynamics of circulating ILCs. ILCs subsets differentially populate various barrier and non-barrier tissues, where they play important roles in tissue homeostasis. Recent findings have provided insight into the mechanisms that guide ILC migration into peripheral tissues, the impact of tissue-specific cues on ILC migration. As such, lymphoid tissue-homing receptors (HR) or trafficking receptor switch is one of the important local immunological milieus. It has been reported that ILCs undergo HR switch, where splenic ILC3 that highly express CCR7, when activated with IL-7 and retinoic acid downregulated CCR7 but upregulated CCR9 and α4β7 expression resulting to an efficient migration to the intestine^100,101^. Interestingly, we found several upregulations of chemokine receptors and ligands in the gut inflammatory ILC3s that highly relates to the incidence of atopic dermatitis^102,103^.

To the best of our knowledge, no reports have indicated a trafficking of intestinal ILCs to skin. We observed that when the reporter KikGr mice were breastfed by milk high in the LCSFA (PA), the intestinal derived red-converted ILCs appeared with greater frequency on the dorsal skin. It is therefore tempting to speculate that the migration of ILCs from the gut to skin may play an important role in the initiation of inflammation in the skin. Another interesting finding is that we suspect that these PA milk induced inflammation or circulatory inflammatory ILC3s may possibly particularly only affect the skin as there were no significant inflammation observed in the other organs, such as the liver, spleen or lung.

It is of consensus that newborn babies require essential nutrients and growth factors from mother’s milk for the shaping and development of the immune system. However, we believe that early exposure to DAMPs, particularly long-chain saturated fatty acids may have a negative impact starting from the intestinal immunity that instigate to increase the risk of atopic dermatitis or possible other allergic or autoimmune diseases. Certainly many murine models of AD have been developed as tools for understanding AD pathogenesis. A comparison of murine models with the human AD transcriptomic profiles have been made, which addresses the complexity of translating murine model to human inflammatory disease^104^. They claimed that IL-23–injected mice model appears to best replicate the human AD profile. IL-23 could drive inflammatory ILC3 activation, and increased IL-23 expression can promote development of pathogenic ILC3s in the newborn intestine (IL-23-ILC3s axis)^105,106^. Their results may be good agreement with our present data suggesting that ILC3 may be potential triggers or worsening factors of AD. As such, the increase of inflammatory ILC3s in our mice model and how it relates to the human counterpart are particularly interesting to be elucidated. The “atopic march” describes the tendency for atopic eczema as the first manifestation of atopy to precede the development of food allergies, asthma, and allergic rhinitis in a temporal sequence^107^. Although it is just a scratch of surface from the complexity of allergies and much further work is required, we hope that our findings will not only lead to a better understanding of the mechanisms of AD and food allergy but ultimately the development of novel strategies and methods to prevent progression of allergies.

## Methods

### Cohorts

A prospective birth cohort of newborn infants was established in Chiba University Hospital and JFE Kawatetsu-Chiba Hospital, from January 2007 to May 2008^22,46–48^. All participants received a questionnaire after their infants were born. Data on parental allergic disease and various exposures were obtained. The parents answered questionnaires with the main focus on symptoms related to eczema lasting for at least two months when the infant was six months of age (first cohort). The complete response rate on the six-month questionnaire was 68%. The diagnosis of atopic dermatitis was carried out by Pediatrician/Allergist with based on the guidelines provided by the Japanese dermatology association (second cohort)^45^. First cohort involved 900 individuals of which only first milk was taken. Second cohort involved 268 mothers with breast milk taken at three sampling points; first milk, 1 month and 6 months. The frozen stock of these mothers' milk was subjected to DAMPs assay (IL-1β ELISA). All participants provided written informed consent to participate in the study. This study was approved by the Human Study Ethics Committee of Chiba University. This Research involving human research participants have been performed in accordance with the Declaration of Helsinki.

### Sampling and storage of mothers’ milk

Breast milk specimens (whole milk collection) were obtained within four days after birth (colostrum), at 1 month and 6 months, and were immediately frozen (− 80 °C) for long-term storage to avoid lipopolysaccharide (LPS) contamination^88^. Milk samples were thawed immediately before testing and were centrifuged (10,000× *g*, 10 min) to obtain surface lipid, interface liquid, and cell-containing pellets. For milk collection from mice, lactating mice were separated from offspring in the same cage with a divider for 3 h prior to milking. Mice were administered with 3 IU of oxytocin (Sigma) via i.p. and within 1 min after oxytocin injection, milk collection was performed using a milking apparatus (KN-591, Natsume Tokyo, Japan) attached to an aspirator.

### Materials and reagents

MSU, phorbol 12-myristate 13-acetate (PMA) and free fatty acids (palmitic acid, palmitoleic acid, myristic acid, myristoleic acid, stearic acid, oleic acid, arachidic acid, arachidonic acid) were obtained from Sigma-Aldrich. Caspase inhibitors Z-VAD-FMK, Z-IETD-FMK, Z-LEHD-FMK were purchased from Calbiochem. Short interfering RNA human PYCARD and anti-rabbit human PYCARD were purchased from Invitrogen and Cell Signaling Technology.

### Cell culture and assays

The human monocytic cell line THP-1 (*ATCC* TIB-202™) was maintained in RPMI 1640 media supplemented with 10% (v/v) foetal bovine serum (FBS; Gibco). Differentiation of monocyte cells towards macrophage-like adherent cells was achieved by treatment, followed by 48 h incubation in untreated cell culture media before experimental assays. Harvesting of PMA-treated cells was achieved by either gentle scraping or by treatment with trypsin/ethylenediaminetetraacetic acid (EDTA; Gibco) for 5–10 min. For the induction of differentiation into macrophage-like adherent cells, THP-1 monocyte cells were treated with 50 ng/mL of phorbol 12-myristate 13-acetate (PMA; Sigma) for 48 h in a 24-well plate. For DAMPs assay, differentiated THP-1 macrophages were treated with MSU (100 µg/mL), fatty acids or mother’s milk samples for 10 h at 37 °C. Cell culture supernatants were assayed for IL-1β using the OptiEIA kit from BD Biosciences according to standard manufacturer instructions. For GDNF assays, small intestine extracts were prepared by treatment of minced tissues with cell lysis buffer (Cell Signaling Technology) for 1 h at 37 °C, followed by sonication for 10 min. Quantitation of GDNF in tissue extracts was performed with the GDNF Emax ImmunoAssay System (Promega). Absorbance at 450 nm was read on a plate reader (Avro X3, Perkin Elmer).

### Differential metabolome analysis of maternal milk contents by LC–MS

Maternal milk was extracted with nine volumes of methanol and insoluble materials, including proteins, were removed by centrifugation. Extracts from five mothers with healthy infants and five with AD infants were serially analysed by LC–MS in the same session to exclude instrumental and environmental variability. A total of 5 µL of methanol extract was separated in a hydrophilic interaction mode with a TSKgel Amide-80 (2.0 × 150 mm, 5 µm, TOSOH, Tokyo, Japan) or in reverse phase mode with a C8 (L-column2, C8, 2.1 × 150 mm, 5 µm, CERI, Tokyo, Japan) connected to a LC (LC20, Shimadzu, Kyoto, Japan). For TSKgel Amide-80 column separation, the LC gradient using mobile phase A and B (A: 10% MilliQ water, 90% acetonitrile, 10 mM ammonium formate, B: 10 mM ammonium formate in MilliQ) was as follows: 0–3 min, 100% A; 3–16 min, 0–50% B; 16–35 min, 50% A, 50% B; 35–40 min, 100% A. For C8 column separation, the LC gradient using mobile phase A and B (A: 99.9% MilliQ water 0.1% formic acid, B: 99.9% methanol 0.1% formic acid) was as follows: 0–3 min, 97% A and 3% B; 3–17 min, linear gradient of 97–0% A and 3–100% B; 17–45 min, 100% A. Elution extracts from the columns were serially ionized with an electrospray ionization (ESI) source heated to 250 °C, which works in positive ion mode at 3000 V or negative ion mode at 2700 V. Sheath and auxiliary gases were set to 50 and 15, respectively. The mass spectra were measured by full scan mode from *m/z* 50 to *m/z* 800 using an Orbitrap Velos Pro or Orbitrap XL mass spectrometers (Thermo Scientific). We used lock mass function of the machines to keep the mass accuracy of the data by detecting continuously appearing peaks of environment origin: for positive ion mode, *m/z* 391.2843 of bis(2-ethylhexyl)phthalate [M + H]^+^ or *m/z* 445.1200 of dodecamethyl cyclohexasiloxane [M + H]^+^ were used, and for negative ion mode, *m/z* 255.2330 of palmitic acid [M-H]^−^ and *m/z* 283.2643 of stearic acid [M-H]^−^ were used. For quantification of fatty acids at negative ion mode, lock mass function was not used unless palmitic acid and stearic acid peak information would be lost. For quantitative analysis of determining the free fatty acid concentration, purchased compounds with serial dilution were separated in a C8 column and analysed at negative ion mode. Peak area of corresponding fatty acids was used to draw standard curves. Content of the fatty acids in the milk samples were calculated using these curves.

### Data analysis

Analysis of mass spectra was performed on MZmine2 (http;//mzmine.github.io/). Ion peaks with high signal intensity (> 10^4^) were extracted from the spectra of every scan, and ion chromatograms of every ion peak with same *m/z* were constructed. Then, chromatogram deconvolution was performed to construct peak lists with *m/z* and retention time. The peak lists of healthy and AD milk samples were aligned with *m/z* and retention time. The aligned peak lists were exported to Microsoft Excel to make further statistical analyses. Then, peak areas of samples were used for multiple comparisons between healthy and AD samples using t-test module of Multiexperiment Viewer (MeV) v4.9 (Dana-Farber Cancer Institute, Boston, MA, USA, http://www.tm4.org/mev.html). with Welch’s approximation for variance assumption and significance determined by *P*-values less than 0.05. PCA (Principle Component Analysis) and OPLS (Orthogonal Partial Least Square projections to latent structures) were performed by utilizing SIMCA-P+ version 14 (Umetrics, Umea, Sweden).

### Molecular Ion identification

Differential ions among samples were further used for molecular identification. Accurate mass value of the peaks was used to list candidate formulae within 3 ppm mass tolerance using QualBrowser of Xcalibur software (Thermo Scientific: version 4.3; (https://www.thermofisher.com/jp/en/home.html).

The human metabolome database (HMDB), http://hmdb.ca/ and KEGG database (https://www.genome.jp/kegg/) was used to identify molecules with candidate formulae. Further confirmation was performed by MS/MS analyses. MS/MS spectra of peaks were compared to the MS/MS data registered in the MassBank (http://www.massbank.jp) and m/z cloud database (https://www.mzcloud.org/). Some candidate molecules were confirmed by the LC retention time and MS/MS spectra of authentic compounds.

### Mice and diet

HR-1 hairless mice were purchased from Hoshino Experimental Animal Centre (Yashio, Japan) *rag-1* KO mice were purchased from The Jackson Laboratory. All mice were kept in a specific-pathogen-free facility in the animal facility of Hiroshima University. Experiments and procedures were performed in accordance with the Institutional Animal Care and Ethics Review Committee for Animal Experimentation of Hiroshima University.

Animals had free access to food and water and were maintained on standard pellet diet (AIN-93G, Research Diets, Inc.) unless otherwise indicated. For the experiments of breast milk feeding, pregnant mice were given a powdered chow (D10012G, Research Diets Inc.) as normal diet. For groups designed as PA milk and OA milk, immediately after birth, powdered chow was changed to a diet high in saturated or high in unsaturated fatty acids by supplementing with an additional 8% (w/w) of palmitic acid (PA, Tokyo Chemical Industry Co. Ltd, Tokyo, Japan) or 8% (w/w) of oleic acid (PA, Tokyo Chemical Industry Co. Ltd, Tokyo, Japan) to the normal diet. Food was returned to normal diet when offspring mice started to wean and feed on their own. The casing of the food and cages were designed to enable access only by the lactating mothers but not the pups.

### Skin analysis and dermatitis scoring

The scoring of dermatitis was evaluated at 15 weeks post-weaning based on the development of 1) erythema/haemorrhage, 2) scarring/dryness, 3) oedema, 4) excoriation/erosion. The skin severity score was defined as the sum of the individual scores as 0 (none), 1 (mild), 2 (moderate), and 3 (severe). Skin moisture and transepidermal water loss(TEWL) were determined using the Delfin MoistureMeterSC and Delfin VapoMeter, respectively.

### Gut and skin lymphocytes isolation

The IEL and LP lymphocytes were isolated and collected separately. Briefly, small intestines were removed from mice, flushed of luminal contents and trimmed of excess fat and connective tissue. The small intestines were opened longitudinally, washed in cold phosphate buffered saline (PBS) and cut into smaller pieces (0.5 cm). Intestinal pieces were then incubated in HBSS media containing 5% FBS, 0.145 mg/mL of EDTA (Sigma) and 5 mM of DTT (Sigma) at 37 °C on a rotating shaker (200 rpm) for 30 min. After incubation, intestinal pieces were gently vortexed for 5 s and supernatants were collected into a separate 50 mL conical tube through a 70 µm strainer. Cell pellets were obtained by centrifugation, suspended in 7 mL of 30% Percoll (GE healthcare) and slowly dispensed onto a layer of 3 mL of 70% Percoll. Percoll gradient centrifugation was performed at 1,500 rpm at 20 °C for 20 min. IEL at the interphase layer of Percoll gradient centrifugation was collected, washed and suspended in FACS buffer containing anti-Fc receptor monoclonal antibody (mAb). Viable cells were counted with trypan blue staining. LP lymphocytes were prepared by digestion of finely minced intestinal pieces remaining after IEL isolation with RPMI 1640 media containing 5 mg/mL of collagenase type IV (Sigma), 1 mg/mL of Dnase I (Sigma), MgCl_2_ (Sigma) and CaCl_2_ (Sigma) in a 37 °C shaker at 200 rpm for 50 min. After digestion, suspension was collected through a 70 µm, then a 40 µm strainer and further enriched by centrifugation over a 40% Percoll gradient. Cell pellets were washed and suspended in FACS buffer containing anti-Fc receptor mAb and counted for viability. For lymphocyte isolation from skin, epidermal and dermal tissues were separated mechanically after 2 h of incubation with 0.15% trypsin–EDTA (0.27 mM) at 37 °C. Tissues were finely minced with scissors and collected with complete media, washed and filtered through a 40 μm strainer. This was repeated two times, pooling all released cells. For dermis, minced tissues were treated with collagenase (sigma) and Dnase I (sigma) for 40 min at 37 °C. Cell pellets were suspended in FACS analysis buffer for antibodies staining and FACS analysis.

### Flow cytometry

For intracellular staining, isolated gut and skin lymphocytes were counted for viable cells (1 × 10^6^) and stimulated with PMA (50 ng/mL) and ionomycin (1 μg/mL) for 3 h at 37 °C. Cells were further incubated for 2 h with Golgi stop solution (containing Monesin, final concentration 1 µM), washed with PBS, before surface antigens staining with Thy1.2 (BD, Pharmingen, Clone: 30-H12) and streptavidin conjugated linear markers; NK1.1 (Biolegend, Clone PK136), TCRγδ (BD pharmingen, Clone: GL3), CD11b (BD Pharmingen, Clone: M1/70), CD8α (BD Pharmingen, Clone: 53–6.7), CD19 (ebioscience, Clone: eBio1D3), GR-1 (BD, Clone: RB6-8C5), and Ter119 (BD Pharmingen, Clone: Ter-119) at 4 °C for 15 min. Cells were then incubated with fixation solution (Cytofix, BD) at 4 °C for 50 min, washed twice and stained with intracellular antibodies; RORγt (BD Horizon, Clone: Q31-378), Gata3 (BD Pharmingen, Clone: L50-823), IL-5 (Biolegend, Clone: TRFK5), IL-13 (ebioscience, Clone: eBio13A), IL-17A (Biolegend, Clone: TC11-18H10.1), and IL-22 (Biolegend, Clone: Poly5164) antibodies at 4 °C in the dark for 30 min. Cells were analysed using FACS Canto II or FACS Fortessa II (BD, Biosciences). Cells sorting was performed using FACS Aria III cell sorter (BD, Biosciences). Flow cytometry data were analysed by FlowJo software (version 10.7.1; https://www.flowjo.com/solutions/flowjo).

### Histological and immunohistological staining

Mice skin and small intestines were fixed in formalin (4% neutral buffered formaldehyde) for 24 h, paraffin-embedded and sectioned at 4 µm thickness. For histochemical staining, sections were deparaffinized and rehydrated through xylene and serial dilutions of ethyl alcohol to distilled water. Haematoxylin–eosin (HE) and toluidine blue staining was performed according to the standard procedure. Frozen sections were prepared by standard cryostat procedures. Specimens were stained with Oil Red O and counterstained with haematoxylin. For immunohistological staining, antigen retrieval was performed by trypsin treatment at room temperature for 20 min. For detection of IgE, sections were incubated with AP conjugated anti-IgE mAb (SouthernBiotech) at 4 °C overnight, followed by DAKO’s fuchsin substrate chromogen system and counterstained with HE or toluidine blue. Immunohistochemistry analysis of IL-17A pAb (abcam, ab91649), IL-22 pAb (abcam, ab18499) was developed using the HRP/DAB (ABC) detection IHC kit (abcam, ab64261) Images were taken with BZ-9000 BioRevo (Keyence) microscope at 100X magnification. Combinations of primary and secondary antibodies used for immunofluorescence were as follows: IL-1β pAb (Invitrogen, P420B), NLRP3 mAb (Invitrogen, PA5-20,838), TSLP pAb (Invitrogen, PA5-20,321), IL-33 pAb (Invitrogen, PA5-20,398), RORγt (Invitrogen, PA5-23,148), Bodipy FL C16 (D3821), Alexa Fluor PLUS 488 (Invitrogen, A32731), and CF660R (Biotium). Slides were mounted with ProLong Diamond Antifade Mountant with DAPI (Invitrogen). Immunofluorescence images were taken with Axiovert 200 fluorescence microscope (Carl Zeiss).

Quantification of immunofluorescence and Oil-Red O staining images were performed by image-j software^108,109^. (version 1.53 ; https://imagej.nih.gov/ij/index.html).

### Quantitation of immunoglobulins and cytokines in serum

Blood sampling of mice was collected via cardiac puncture, and serum was obtained by centrifugation after incubating whole blood for 30 min at 37 °C. Immunoglobulin isotyping of blood sera was performed using the mouse immunoglobulin isotyping panel FlowCytomix 6plex Kit and IgE FlowCytomix Simplex Kit (eBioscience). Immunoglobulin titres were calculated using the FlowCytomix Pro Analysis software (eBioscience). For OVA-specific IgE analysis, mice at the age of 5 weeks were immunized with 10 μg Ovalbumin in 1 mg of alum adjuvant via intraperitoneal injections. The second and third boost of OVA-alum were administered at one-week intervals consecutively from the initial immunization. Blood sera was sampled at three intervals, before initial immunization, before second boost and one week after the second boost. OVA-specific IgE levels in serum were determined using the DS mouse IgE ELISA (OVA) (DS Pharma Biomedical). Cytokine levels were detected using the Mouse Th1/Th2/Th17/Th22 13plex Flow Cytomix Kit (eBioscience) according to the manufacturer’s protocol. Cytokine data were obtained by flow cytometry (FACS Calibur, BD Biosciences) and analysed using the FlowCytomix Pro Analysis software (eBioscience).

### Adoptive transfer and KikGr mice

Skin-to-skin adoptive transfer was performed by isolation of skin lymphocytes from donor mice and cell suspension prepared in PBS were directly injected into the dorsal skin of recipient mice at random spots. Adoptive transfer of gut ILCs isolated form donor mice were administered by i.v. injection through the tail of the recipient mice. Isolated gut ILCs were incubated with Qtracker 525 cell labelling (Invitrogen) for 1 h at 37 °C prior to i.v. into recipient mice. Qtracker-labelled cells on the dorsal skin of recipient mice was detected using an in vivo imaging system (NightOwl LB983, Berthold technologies). Transgenic KikGR mice were obtained from the RIKEN Bio Resource Centre. Survival surgeries were performed with proper anaesthetic and sterile technique. Mice were anaesthetised with isoflurane [1–3% (v/v) by inhalation] and the fur around the abdominal area was shaved and disinfected with ethanol before incision. The cecum was identified and the small intestine was gently externalized and localized of onto a towel with a piece of sterile foil underneath and moistened with saline. Peyer patches were covered with small foil strips, as well as the other body parts to shield from the exposure of UV light. The small intestine was irradiated with violet light at both sides (150 mW/cm^2^, 50 s) using spot UV-curing equipment (SP11; USHIO) fitted with a 436 nm g-line band-pass filter and a fiber optic cable (SF101-NQ). Light intensity was measured with a UIT-250 digital photometer (UVD-S405 detector; USHIO), and light source distance was set accordingly. After final irradiation, the abdominal cavity and skin were closed with 5–0 nylon suture. Analysis of KikGR immunofluorescence was observed with the in vivo imaging system (NightOwl LB983, Berthold technologies) and the ratio of photoconverted red to green fluorescence on the shaved dorsal skin was analysed with IndiGO software (version 2.0.5.0; https://www.berthold.com/en/bioanalytic/products/in-vivo-imaging-systems/nightowl-lb983/).

. Detection of fluorescence was performed by the settings of excitation at 470 nm for green, 530 nm for photoconverted red, respectively; and emission signals at 520 nm and 600 nm, respectively. Epidermis from the post-surgery KikGR mice were isolated, stained with antibodies for lineages, intracellular RORγt and analysed by FACS (BD LSRFortessa).

### RNA sequencing analysis

IEL and LP lymphocytes were isolated as described in the isolation methodology. ILC3s were sorted via the FACS ARIA III (BD) by cell population gated for lineage negative, CD4^+^, Thy1.2^+^, CD25^+^, and Nrp1^+^. Total RNA was prepared by using the Qiazol lysis buffer and miRNeasy Micro Kit (Qiagen). The purity of samples (RIN) were analysed on the Agilent 2100 Bioanalyzer System and quantified. cDNA libraries were constructed using the SMART-Seq v4 Ultra Low Input RNA Kit (Takara) for amplification and sequencing using standard Illumina protocols. Briefly, cDNA fragmentation was carried out by the CovarisS2 followed by preparation with the KAPA Library Preparation Kit (KAPA biosystems) and sequenced on an Illumina HiSeq 2500 (TruSeq DNA Sample Prep Kit v2, Illumina). The sequencing run was analysed with the Illumina CASAVA pipeline (v1.8.2), with demultiplexing based on sample specific barcodes. The reads were mapped onto the reference mouse genome mm10 using TopHat ver.2.1.1 in combination with Bowtie ver.2.2.8.0 and SAMtools ver. 0.1.18. Gene expression was quantified with Cufflinks ver. 2.2.1, and analyzed with KEGG pathway database^110^ The data sets are deposited in the Gene Expression Omnibus (GEO) repository under the accession number GSE 124182.

### Fecal sampling and 16S rRNA amplicon sequencing

Pregnant mice were co-housed in order to make their gut microbiota composition become similar before separation for feeding PA, OA, or ND diet feeding. Offspring mice were separated into cages with male and female according to their mother origin and feces were collected.

16S rRNA amplicon sequencing was performed as previously reported^111^. In brief, bacterial genomic DNA was isolated from 5 mg of freshly frozen feces, and PCR amplified V1-2 regions of the 16S rRNA gene was sequenced. The sequences were demultiplexed, quality filtered, and taxonomically assigned against Greengenes data base with RDP-classifier pipeline of the QIIME software package^112^. An operational taxonomic unit (OTU) was defined at 97% similarity. OTUs indicating relative abundance of under 0.1% were filtered to remove noise. The alpha-diversity and the beta-diversity calculated with the unweighted and weighted Unifrac distance were analyzed using the QIIME2 software package *(*version 2017.6; http://qiime.org). Relative abundance data were applied to LEfSe on Galaxy platform^113^ to identify specific gut microbial taxa with significant linear discriminant analysis effect size. Hi bulldozer.

### Statistical analysis for biological experiments

For biological experiment (non-omics) analyses, data were analysed using Prism 8 software (GraphPad, GraphPad Prism 8.4.3 ; https://www.graphpad.com/scientific-software/prism/) by one-way ANOVA with Newman–Keuls’s test. *P* < 0.05 was considered significant.

For gut microbiota analysis, data are represented as mean ± SD. *P < 0.05, **P < 0.01, ***P < 0.001, One-way ANOVA with post-hoc Tukey’s multiple comparison test.

### Ethics

All experiments involving animals were performed in accordance with the ARRIVE guidelines. All animal experimental procedures were approved and authorized by the Institutional Animal Care /Animal Experiments Committee of Hiroshima University and complied with the guidelines for the care and use of laboratory animals at Hiroshima University.

## Supplementary Information

Supplementary Information.

## Data Availability

Raw RNA- sequencing data have been uploaded to the NCBI Gene Expression Omnibus (GEO) database under identifier GSE 124182. Other relevant data are available from the corresponding author upon reasonable request.
