# Supplementary material for: Long-chain saturated fatty acids in breast milk are associated with the pathogenesis of atopic dermatitis via induction of inflammatory ILC3s
Source: Sci Rep. 2021 Jun 23;11:13109. doi: 10.1038/s41598-021-92282-0 (PMC8222289; doi:10.1038/s41598-021-92282-0)
Supplement: Supplementary file 1 — Supplementary Information. [file 41598_2021_92282_MOESM1_ESM.pdf]

Supplementary Information

Supplementary Figs S1 to S13 and Tables 1 and 2

Long-chain saturated fatty acids in breast milk are associated with the pathogenesis of atopic dermatitis via induction of inflammatory ILC3s

Weng-Sheng Kong[1], Naohiro Tsuyama[2,7†], Hiroko Inoue[1], Yun Guo[1], Sho Mokuda[3], Asako Nobukiyo[4], Nobuhiro Nakatani[5], Fumiya Yamaide[6], Taiji Nakano[6], Yoichi Kohno[6††], Kazutaka Ikeda[7†††], Yumiko Nakanishi[8,9,10], Hiroshi Ohno[8,9,10,12], Makoto Arita[7], Naoki Shimojo[6,12],& Masamoto Kanno[1,11,12] *

[1]Department of Immunology, [2]Analytical Molecular Medicine and Devices Laboratory, [3]Department of Clinical Immunology and Rheumatology, Graduate School of Biomedical and Health Sciences, [4] Natural Science Centre for Basic Research and Development, [5]Technical Center, Hiroshima University, Japan. [6]Department of Pediatrics, Graduate School of Medicine, Chiba University, Japan. [7] Laboratory for Metabolomics and [8] Laboratory for Intestinal Ecosystem, RIKEN Center for Integrative Medical Sciences (IMS), Japan. [9] Intestinal Microbiota Project, Kanagawa Institute of Industrial Science and Technology, Japan, [10] Immunobiology Laboratory, Graduate School of Medical Life Science, Yokohama City University, [11]AMED-SENTAN, [12]AMED-CREST Japan Agency for Medical Research and Development.

Correspondence: *mkanno@hiroshima-u.ac.jp

Department of Immunology, Graduate School of Biomedical and Health Sciences, Hiroshima University, 1-2-3, kasumi, Minami-ku, Hiroshima 734-8551, Japan

Keywords: Atopic dermatitis, type 3 innate lymphoid cells, breast milk, DAMPs

a Cohort Study
Atopic dermatitis (AD) development in breastfed infants

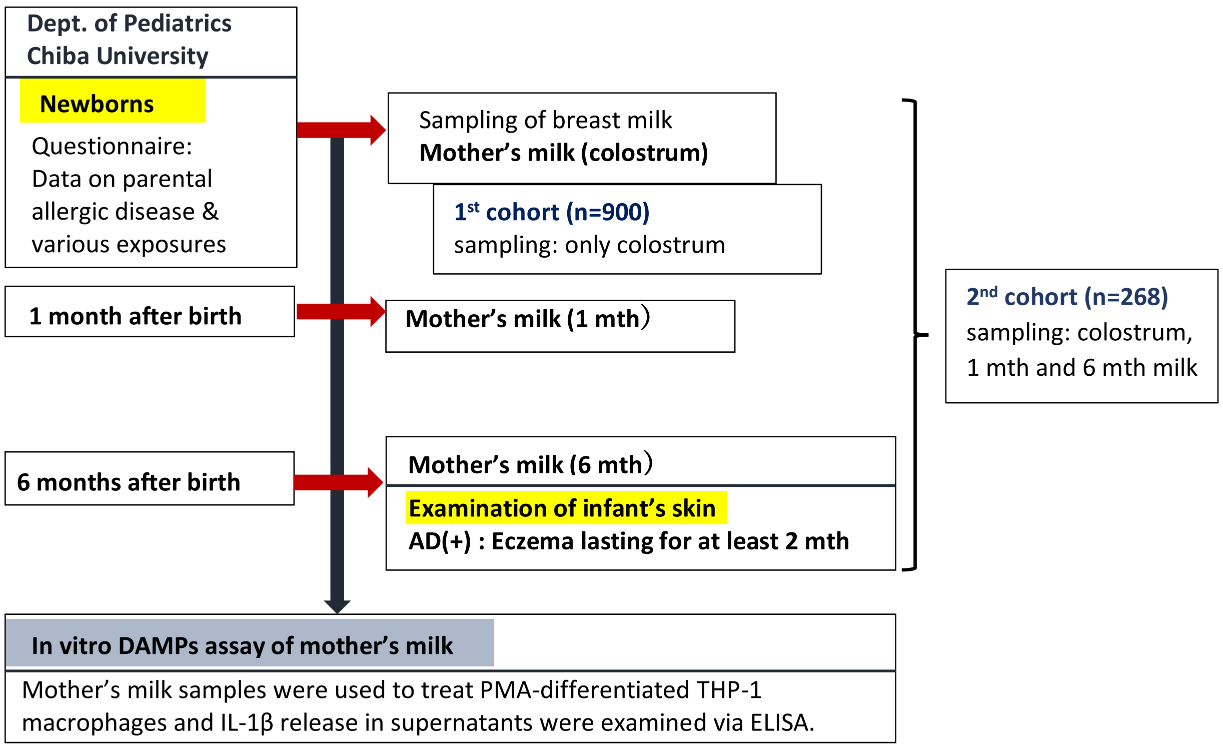

b

Second cohort study (n=268 mothers)				
DAMPs assay (IL-1β release)	Mother's milk samples			
	First milk	Milk (1 mth)	Milk (6mth)	No. of samples
All negative	-	-	-	70
All positive	+	+	+	23
Positive for at least one or more combinations of the sampling time points	+	+	-	175
	+	-	-	
	-	+	-	
	-	-	+	
	-	+	+	
	+	-	+	

c

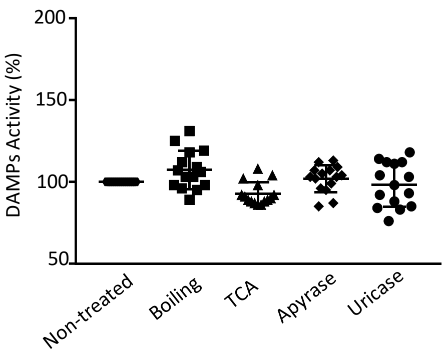

Supplementary Fig S1. Human cohort study reveals DAMPS activity in mother's milk of which infants developed atopic dermatitis. (**a**) Schematic flow chart depicting the collection of mother's milk and DAMPs assay. First cohort study group involved 900 new born infants, in which DAMPs analysis were performed only for mothers' milk collected at first milk. (**b**) Second cohort (n=268) involved analysis of DAMPs activity in mother's milk taken at first milk/ colostrum, 1 month and 6 months after birth. Infants skin condition were monitored and examined for atopic dermatitis at 6 months old and those with eczema lasting for at least 2 months were grouped as atopic dermatitis (AD+). Mother's milk shows ability to induce IL-1β production at least in one or combinations of the sampling time points. (**c**) Measurement of DAMPs activity in mother's milk with pre-treatment as indicated (n=15).

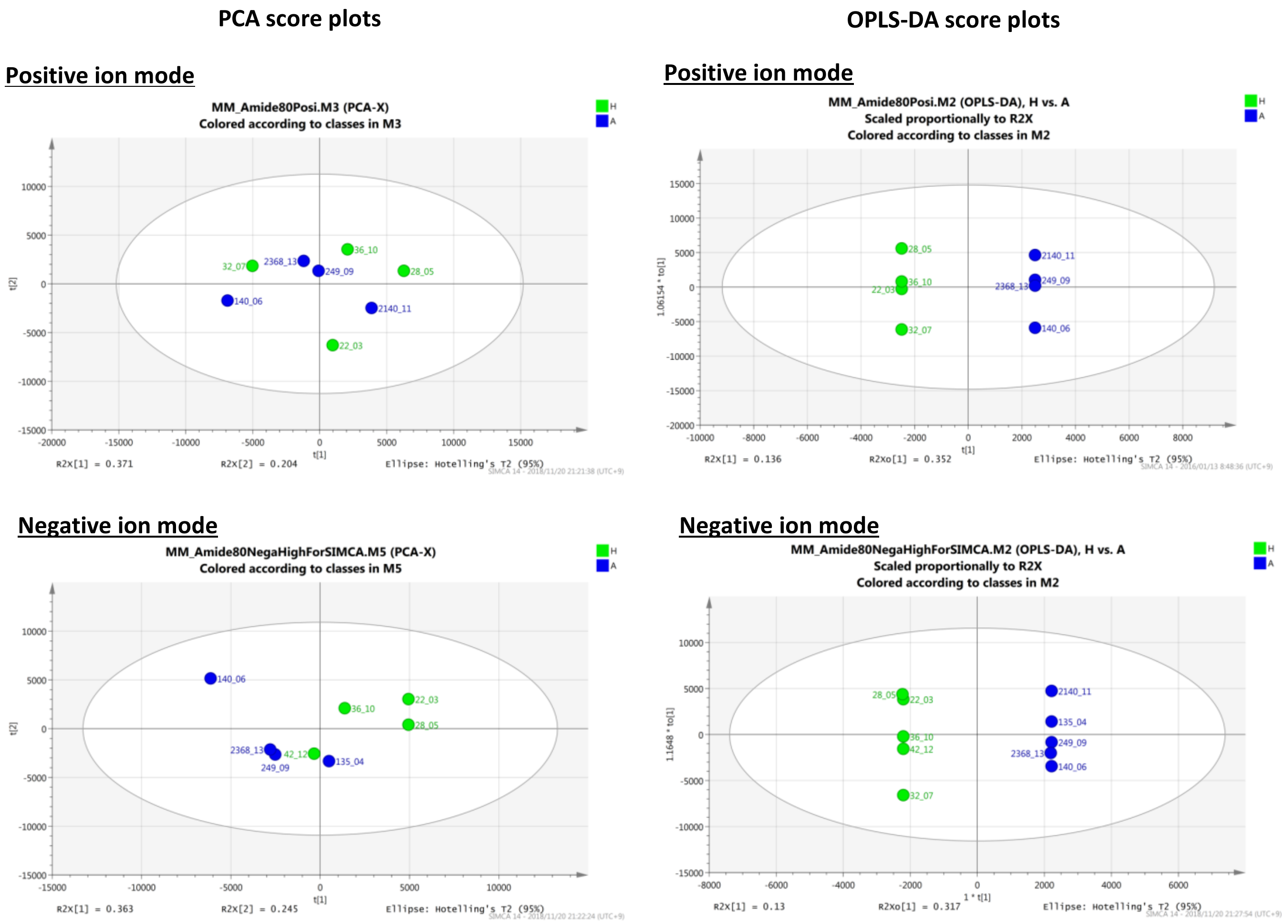

Supplementary Fig. S2. Multivariate analysis of maternal milk metabolome. To figure out the global metabolite similarity among samples, aligned peak list were normalized against total ion count of whole dataset and analyzed by principal component analysis (PCA) using SIMCA version 14. Score plot showed that metabolomic variations between normal and allergy samples were very small especially in positive ion mode, whereas the orthogonal partial least square (OPLS) analyses succeeded to separate the characteristic profile to each group indicating that small differences existed between groups.

a Positive ion mode　　**b** Negative ion mode

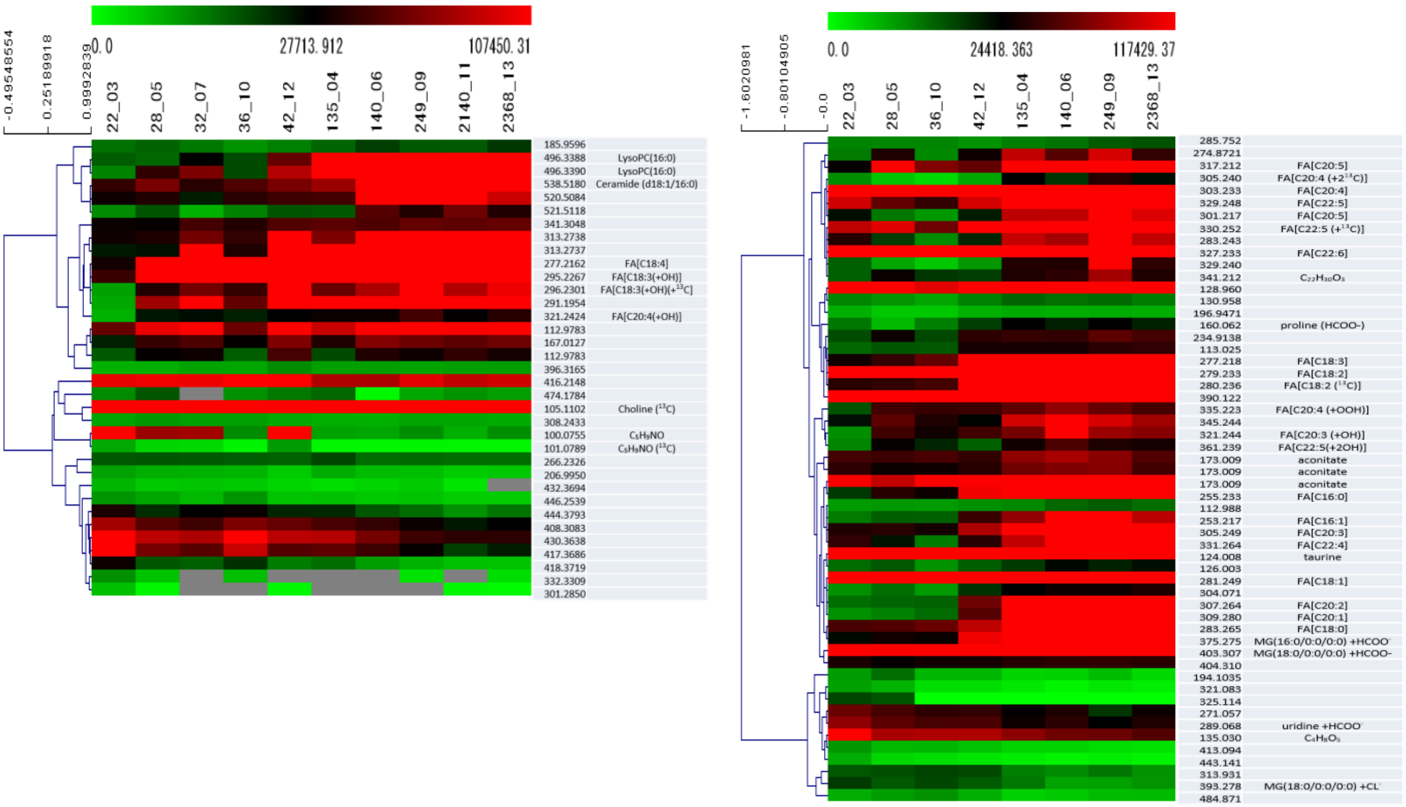

Supplementary Fig. S3. Heat map shows the changes in metabolites between maternal milk with healthy or AD(+) infants. Hierarchical clustering of differential metabolites. (a) positive ion mode and (b) negative ion mode. Differential peaks were applied on hierarchical clustering to draw heat maps using MeV. Correlation of peak values and color are indicated at the top. Red, high and green, low. Sample name are indicated in the top (22, 28, 32, 36, 42: healthy, 135, 140, 249, 2140, 2368: AD(+), numbers followed by sample name indicates the turn of analyses). M/Z and candidate name of every peak are indicated in the right, and sample name. Data are analyzed with Xcalibur (version 4.3 , https://www.thermofisher.com/jp/en/home.html)

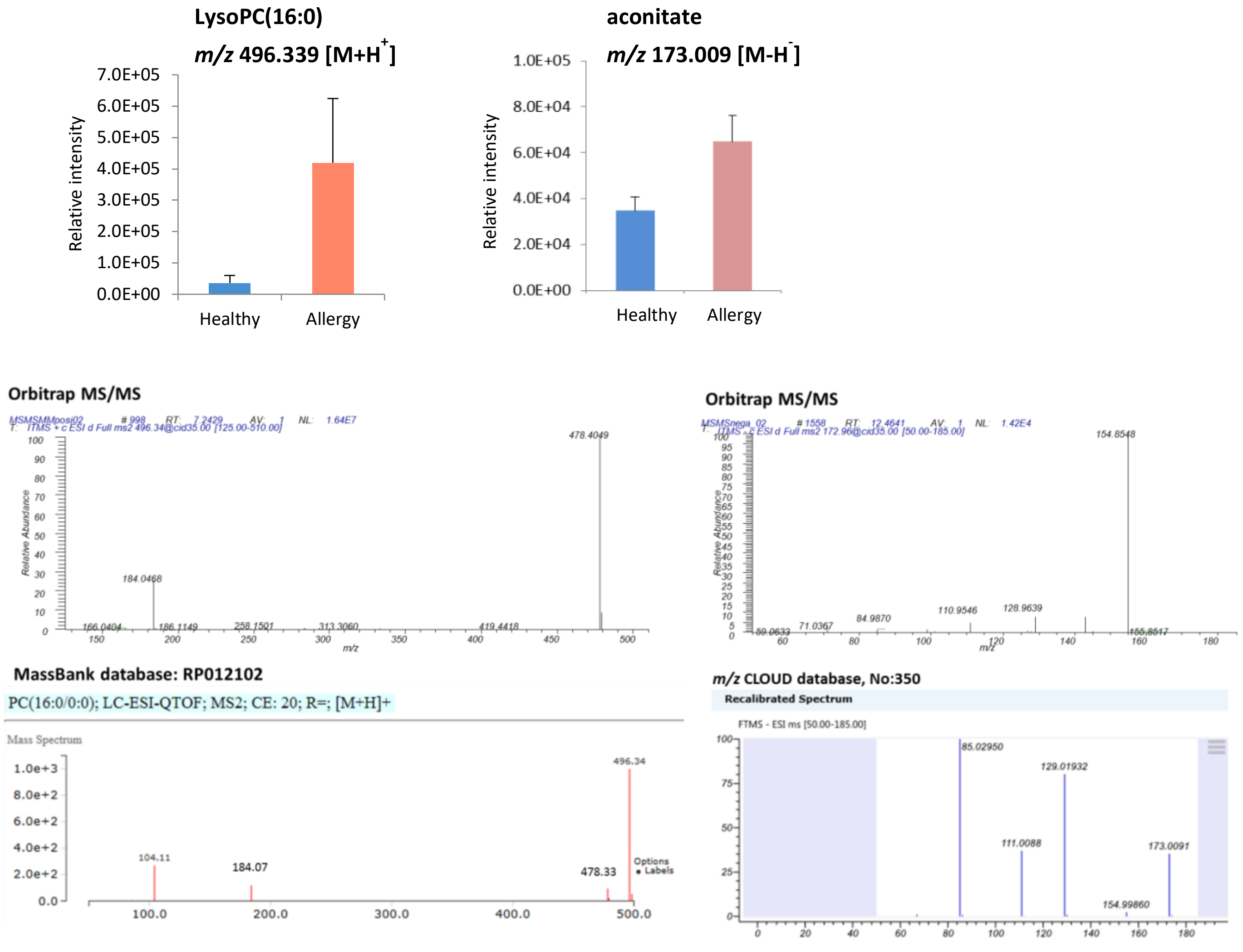

Supplementary Fig. S4. Comparison of MS/MS-identified differential peaks. Peak intensity of LysoPC and aconitate in healthy and allergy (AD+) milks (n=5, error bars indicate +SD). MS/MS spectra obtained from milk extracts are also indicated with the records in the databases (from MassBank for LysoPC and from m/z CLOUD for aconitate. MS/MS identified differential peaks (*P*<0.05).

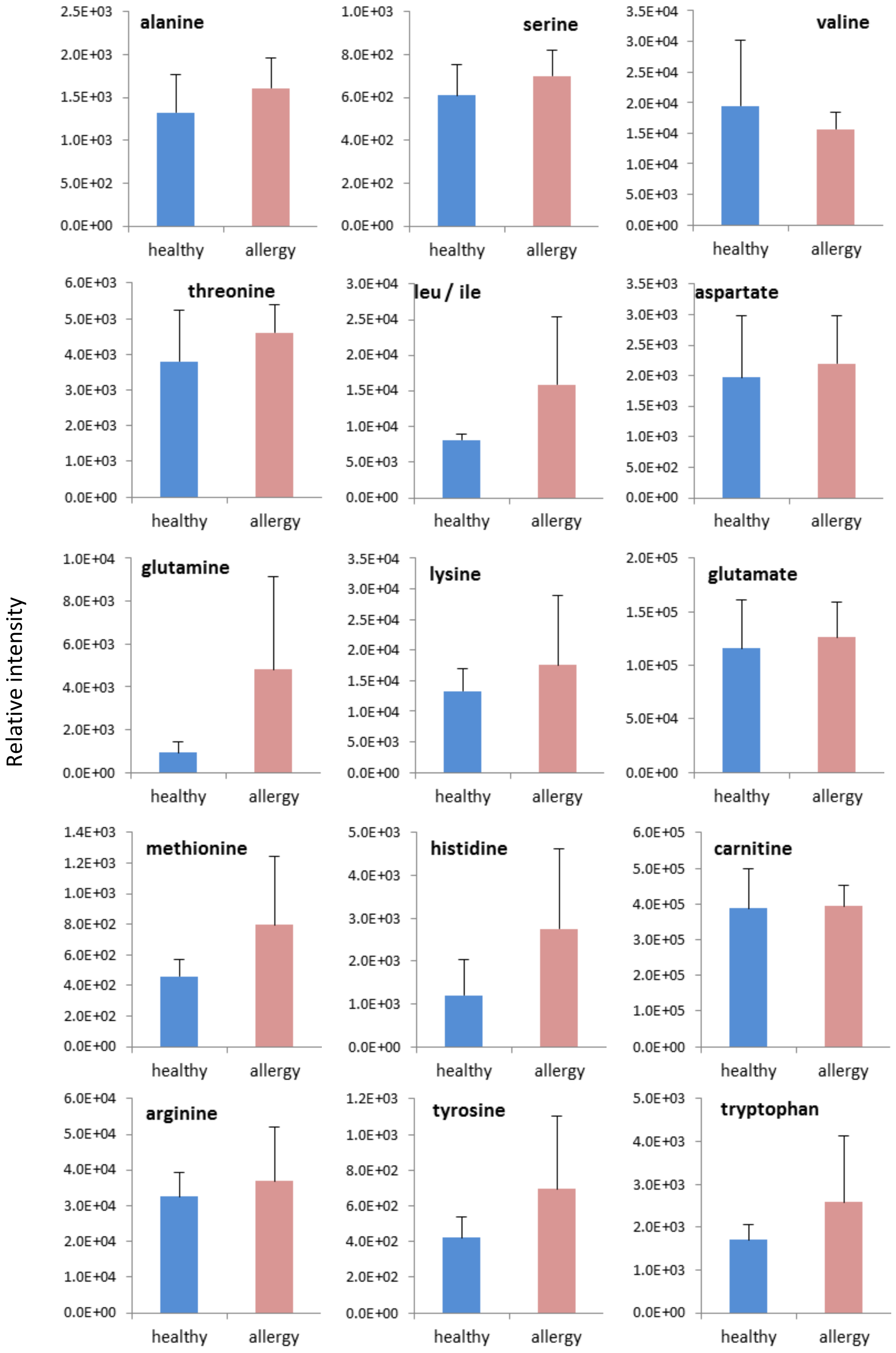

Supplementary Fig. S5. Comparison and relative intensitiy of amino acids analyzed by HILIC column. Difference of indicated amino acid peaks between healthy and allergy (AD+) milks show no statistical significance

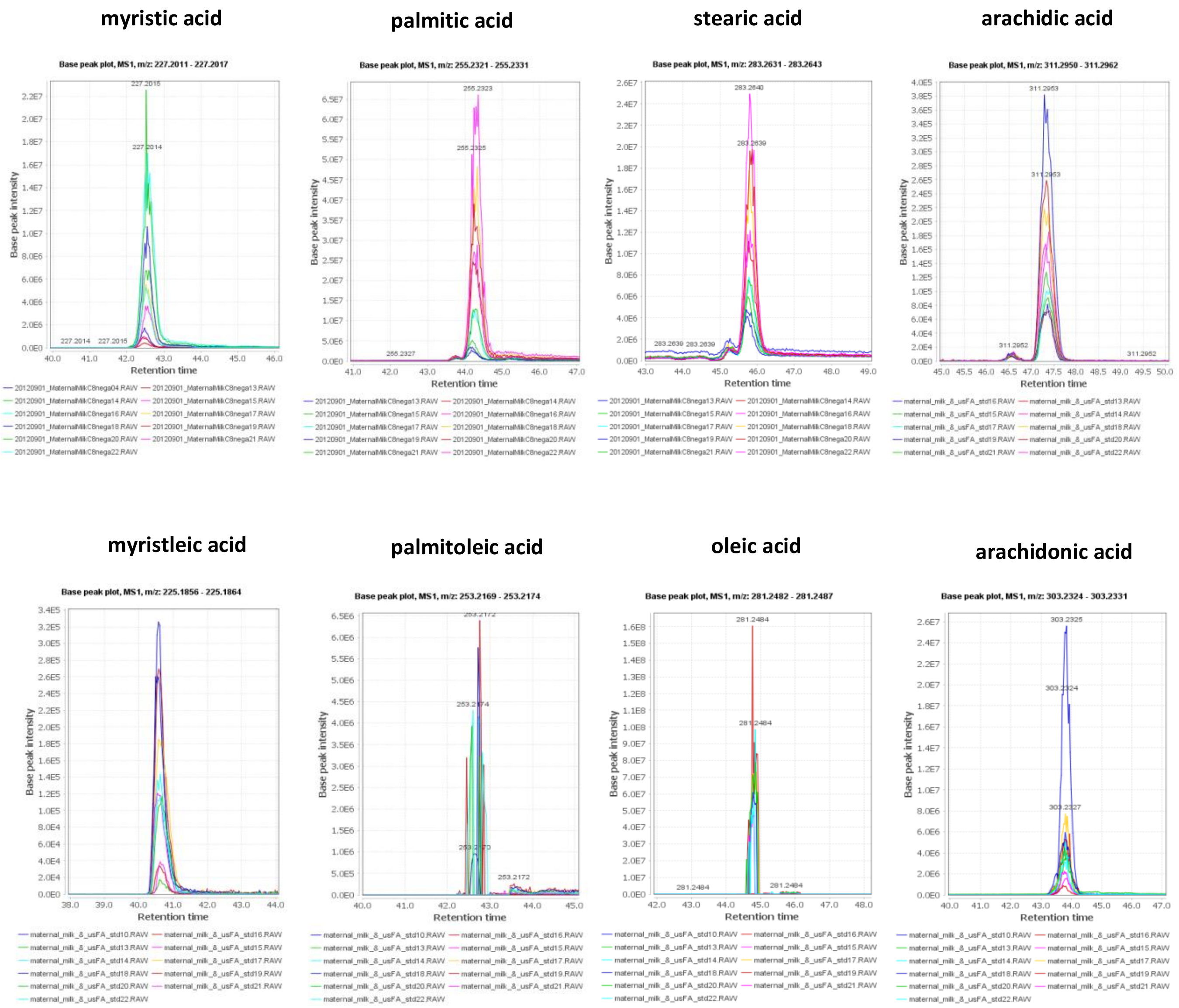

Supplementary Fig. S6. Ion chromatogram of fatty acids in standard material and milk samples. C8-separated ion chromatograms of corresponding fatty acids were indicated.

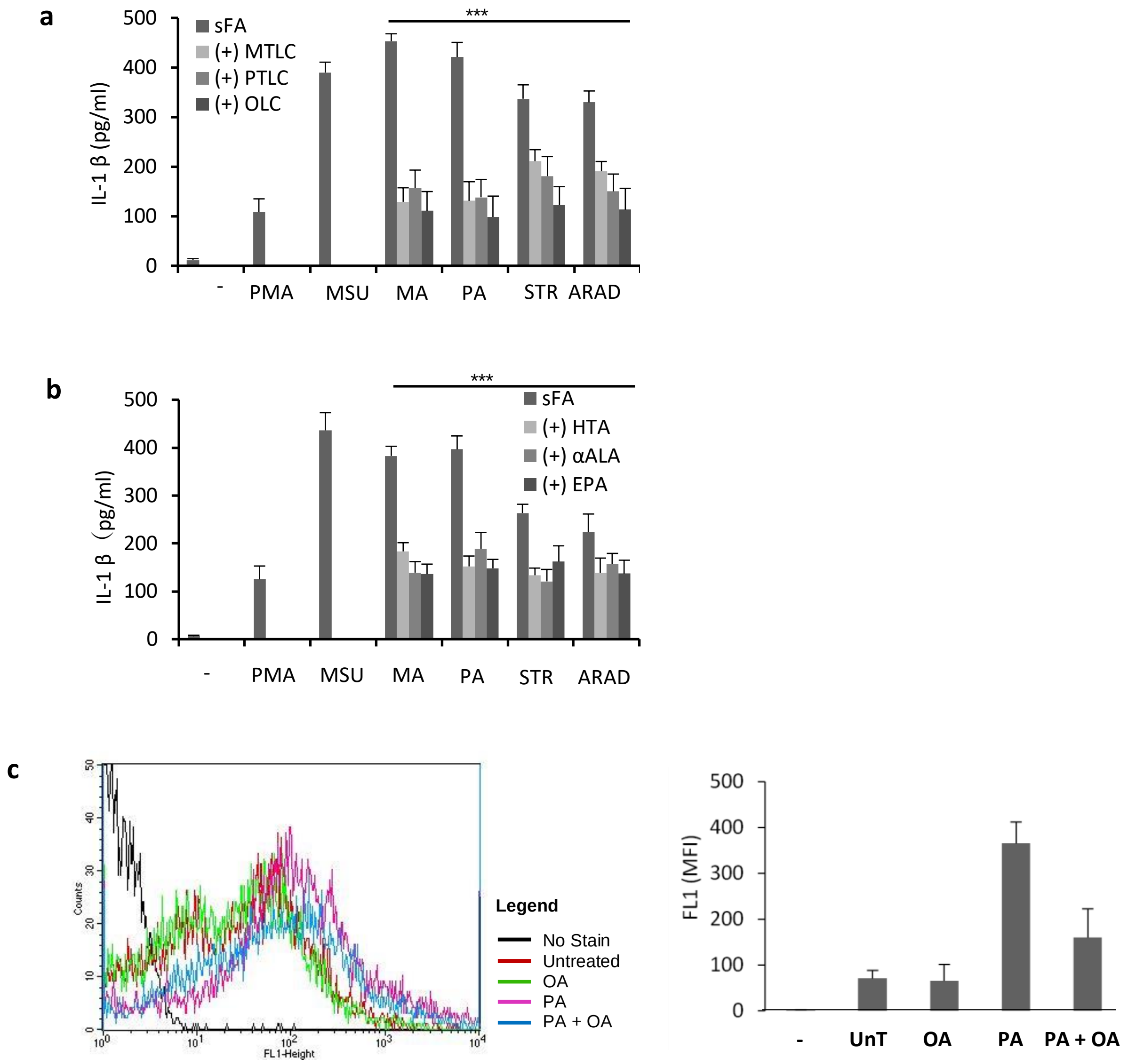

Supplementary Fig. S7. Long-chain saturated fatty acids are potent DAMPs but not long chain unsaturated fatty acids. PMA-differentiated THP-1 macrophages were treated with long-chain saturated fatty acids alone (MA: myristic acid, PA: palmitic acid, STR: stearic acid, Arachidic acid: ARAD, 100 µM) or in the presence of (**a**) long-chain unsaturated fatty acids (MTLC: myristoleic acid, PTLC: palmitoleic acid, OA: oleic acid) or (**b**) omega-3 fatty acids (HTA: hexadecatrienoic acid, ALA: α-linolenic acid, EPA: eicosapentaenoic acid) at 100 µM, respectively. (**c**) Caspase 1 activity of treated cells were measured via flow cytometry using the caspase-1 specific substrate YVHDAP green kit (CaspaLux, OncoImmunin, Inc) and FL1 means are presented as bar graphs (means ± SD). . *$P < 0.05$, **$P < 0.01$, ***$P < 0.001$, one-way ANOVA. Data are analyzed with **FlowJo (version 10.7.1. https://www.flowjo.com/solutions/flowjo/downloads), and Prism 8 (version 8.4.3. https://www.graphpad.com/scientific-software/prism/)**

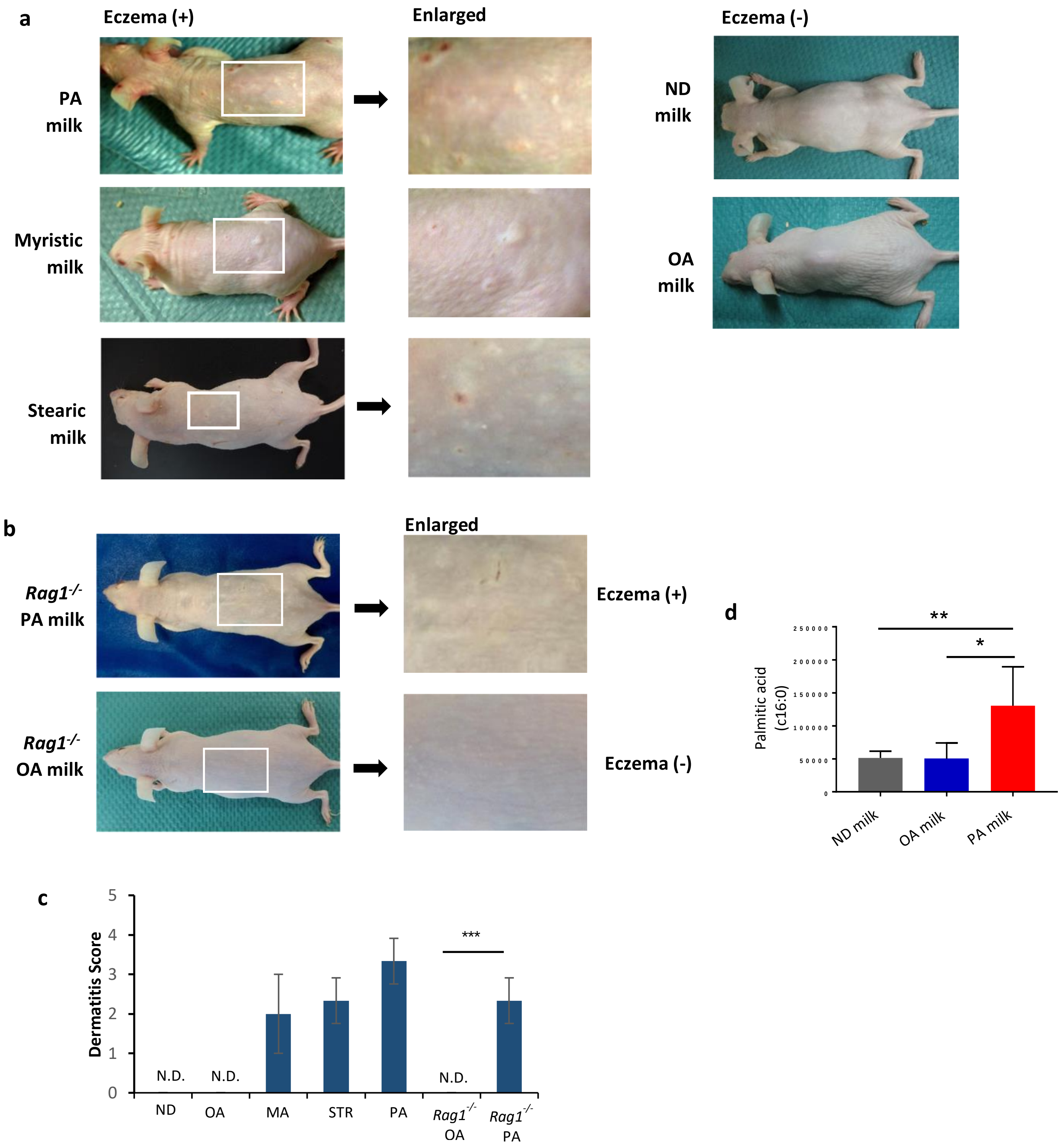

Supplementary Fig. S8. Milk high in long-chain saturated fatty acids increases incidence of atopic dermatitis in mice offspring. (**a**) Mice offspring fed with milk from mothers of high saturated fatty acids diet (palmitic acid, myristic acid and stearic acid) developed skin complications (eczema) at an average of 15 weeks post-weaning (p.w.). (**b**) HR-1 *Rag1* knockout mice were constructed by cross breeding of HR-1 mice to *Rag1*$^{-/-}$ B6 mice for at least 10 generations. Mice offspring of HR-1 *Rag1*$^{-/-}$ fed with milk from mothers of high PA diet developed eczema at an average of 15 weeks p.w. (**c**) Dermatitis score of mice fed by maternal mice of ND or high OA, STR, MA, or PA milk. (ND: normal diet, OA: oleic acid, STR: stearic acid, MA: myristic acid, PA: palmitic acid) All mice of ND, OA, and *Rag1*$^{-/-}$OA did not develop any skin symptoms (dermatitis score O, N.D. not detected). (**d**) Quantitation of palmitic acid (C16:0) in milk taken from lactating mice given diet high in palmitic acid (PA milk), oleic acid (OA milk) or normal diet (ND milk) (n=5 /group). Data are represented as mean ± SD. *P< 0.05, **P< 0.01, ***P < 0.001, one-way ANOVA. Data are analyzed with **Prism 8 (version 8.4.3. https://www.graphpad.com/scientific-software/prism/).**

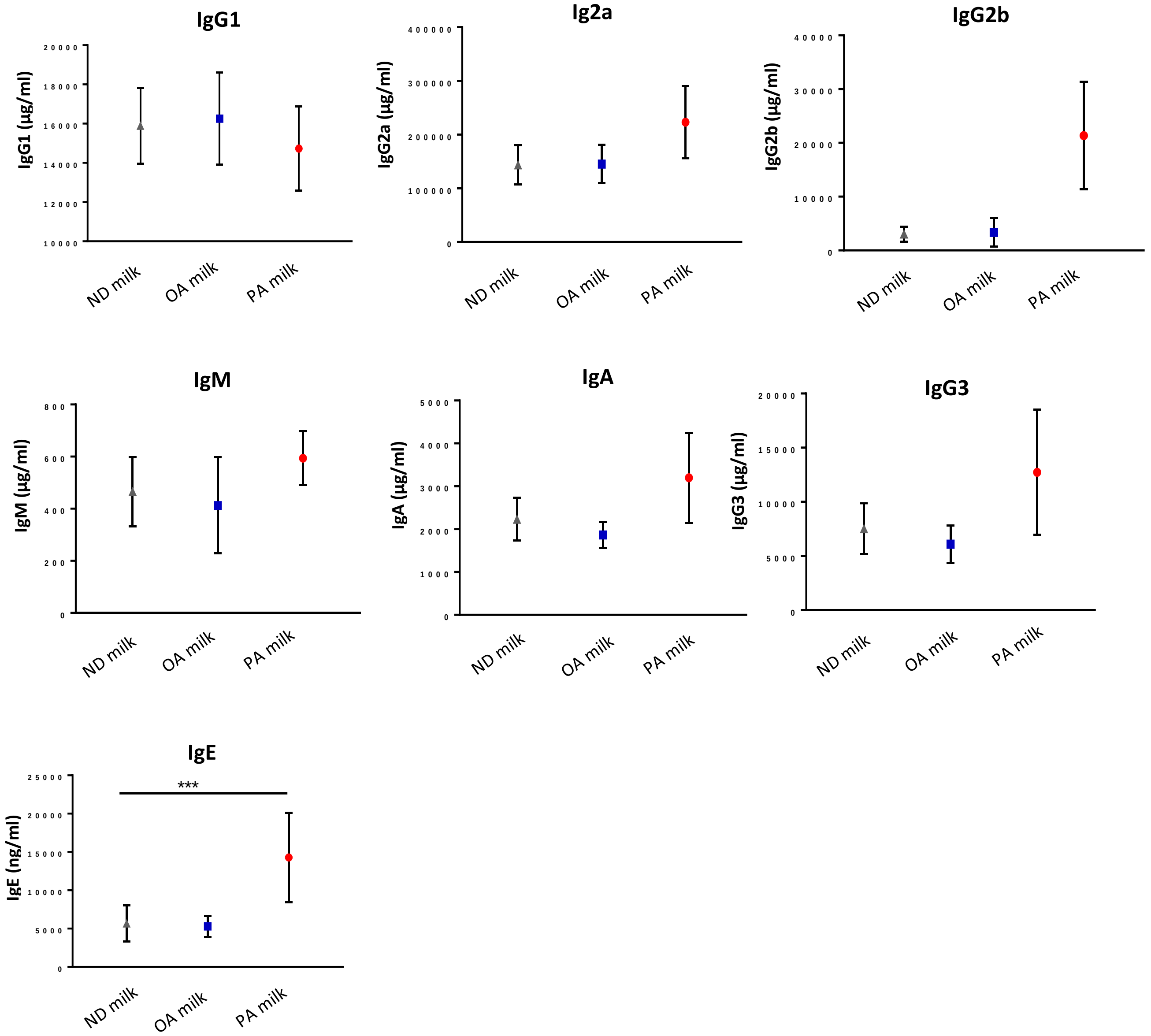

Supplementary Fig. S9. Quantitation of immunoglobulins in blood serum of ND milk, OA milk and PA milk at 15 weeks post-weaning with mouse immunoglobulin iostyping kit (6plex and IgE simplex, eBioscience). . **P* < 0.05, ***P* < 0.01, ****P* < 0.001, one-way ANOVA. Data are analyzed with **Prism 8 (version 8.4.3. https://www.graphpad.com/scientific-software/prism/).**

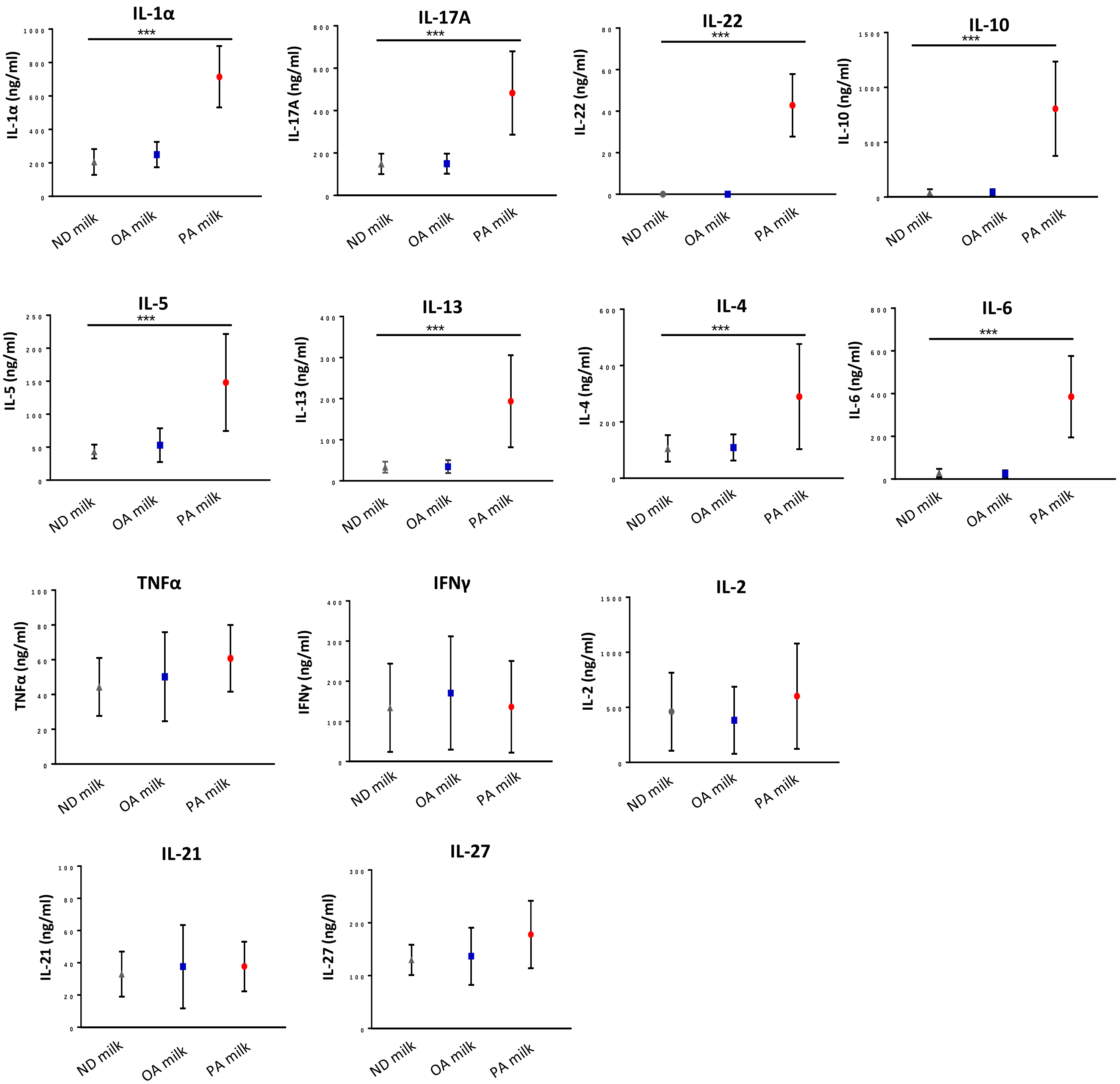

Supplementary Fig. S10. Quantitation of cytokines in blood serum of ND milk, OA milk and PA milk at 15 weeks post-weaning with mouse cytokine panel kit (LEGENDplex^{TM} mouse Th cytokine panel, Biolegend). . *P < 0.05, **P < 0.01, ***P < 0.001, one-way ANOVA. Data are analyzed with **Prism 8 (version 8.4.3. https://www.graphpad.com/scientific-software/prism/).**

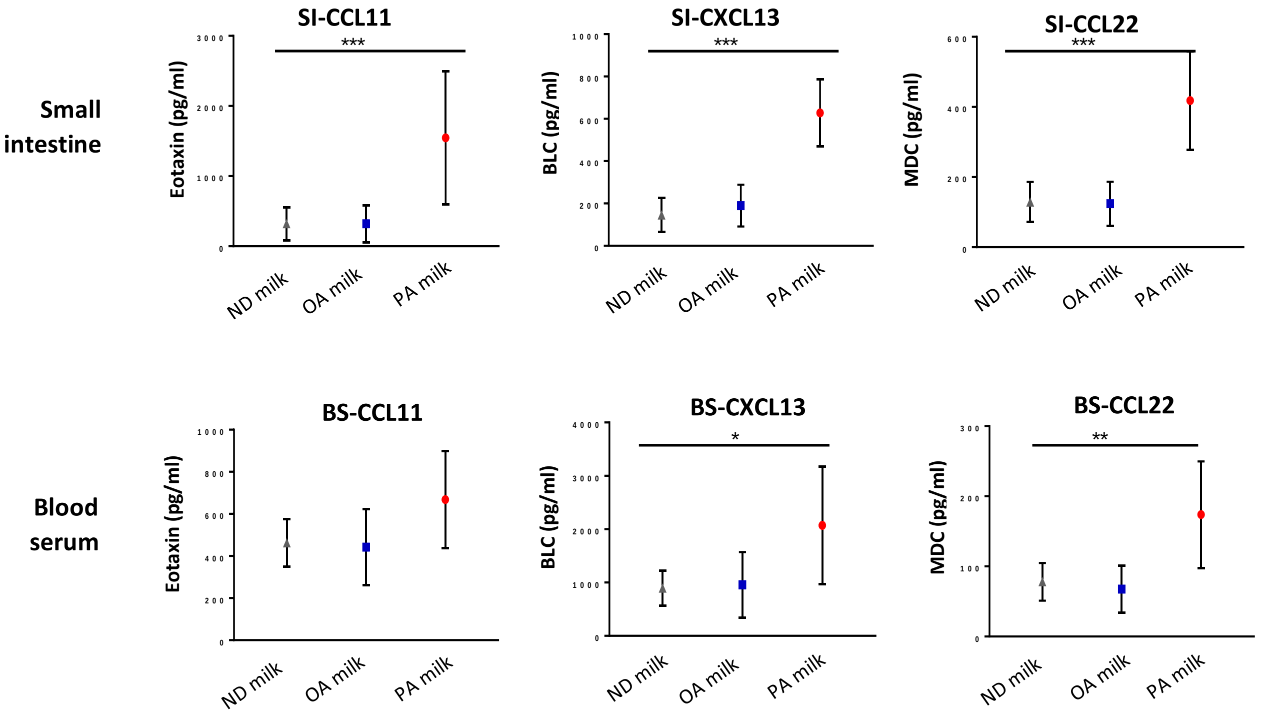

Supplementary Fig. S11. Quantitation of chemokines in small intestine extracts and blood serum of ND milk, OA milk and PA milk at 15 weeks post-weaning with mouse chemokine panel kit (LEGENDplex™ mouse proinflammatory chemokine panel, Biolegend). . **P* < 0.05, ***P* < 0.01, ****P* < 0.001, one-way ANOVA. Data are analyzed with **Prism 8 (version 8.4.3. https://www.graphpad.com/scientific-software/prism/).**

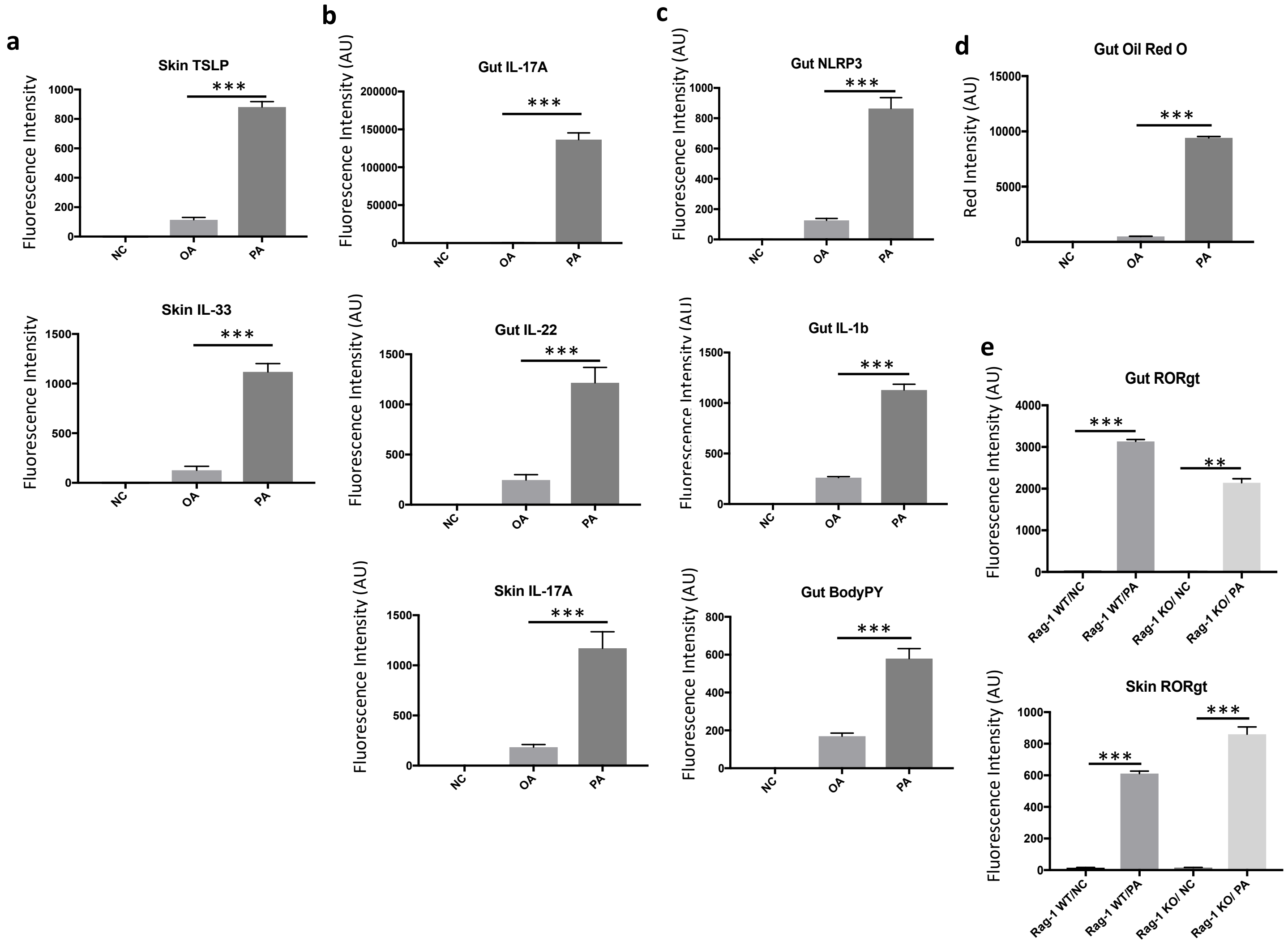

Supplementary Fig. S12

(**a-c, e**) Quantitation of fluorescence intensity by Image-j, in relation to the immunofluorescence staining of intestine and skin (Fig 5B, 5E, 6A, and 7G). (**d**) Quantitation of Oil-Red O stain(Fig 6b) by image-J. Data are represented as mean ± SD. **P* < 0.05, ***P* < 0.01, ****P* < 0.001, one-way ANOVA. Data are analyzed with **Prism 8 (version 8.4.3. https://www.graphpad.com/scientific-software/prism/).**

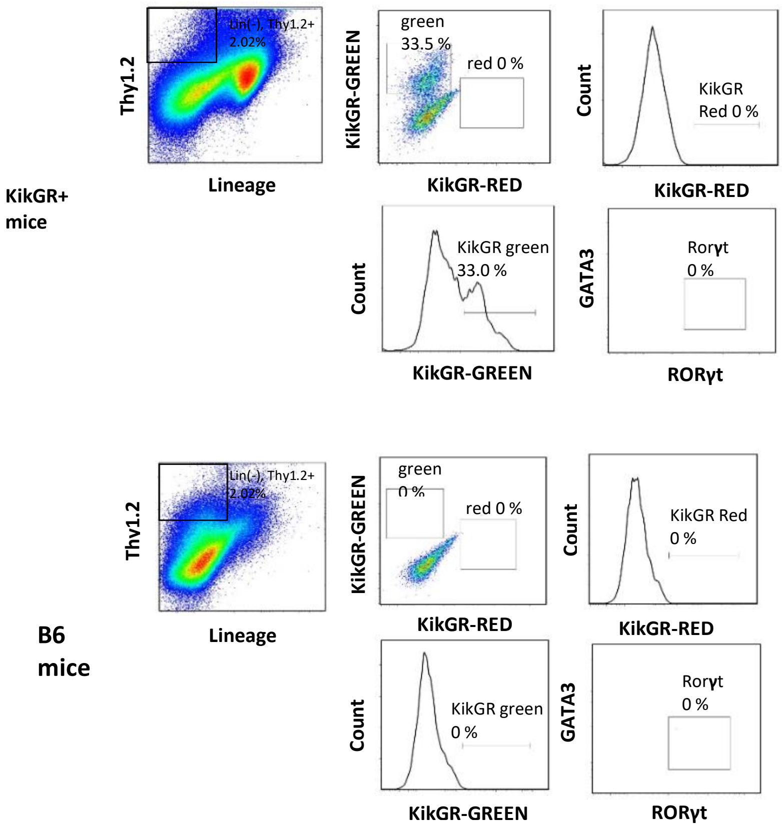

Supplementary Fig. S13. FACS Analysis of fluorescence expression in KikGR positive and B6 mice. Cells from the dorsal skin (dermis) were isolated and intracellularly stained for RORγt. Representative FACS analysis of isolated skin lymphocytes, gated for lineage -, Thy1.2 +, RORγt +(ILC3s), KikGR green and red fluorescence. Data are analyzed with **FlowJo (version 10.7.1. https://www.flowjo.com/solutions/flowjo/downloads)**

Supplementary Tables

Supplementary Table 1: Positive and negative ion mode analysis of milk samples

Positive ion mode

m/z	retention time (min)	fold change (allergy/normal)	candidate name $[M+H]^+$
100.076	1.93	0.10	
101.079	2.06	0.16	C_5H_9NO (^{13}C)
105.110	9.74	0.64	Choline (^{13}C)
112.978	1.37	1.14	
112.978	1.36	1.34	
167.013	1.36	1.31	
185.960	9.99	1.31	
206.995	8.43	0.85	
266.233	1.05	0.94	
277.216	1.17	1.93	FA[C18:4]
291.195	1.38	1.67	
295.227	1.18	1.72	FA[C18:3(+OH)]
296.230	1.18	1.69	FA[C18:3(+OH)(^{13}C)]
301.285	7.13	0.13	
308.243	2.38	0.89	
313.274	1.04	2.86	
313.274	1.14	3.04	
321.242	1.17	1.61	FA[C20:4(+OH)]
332.331	1.53	0.26	
341.305	1.04	1.40	
396.316	3.12	1.05	
408.308	1.11	0.58	
416.215	1.20	0.81	
417.369	1.09	0.47	
418.372	1.10	0.46	
430.364	1.10	0.53	
432.369	1.10	0.54	
444.379	1.11	0.60	
446.254	1.43	0.64	
474.178	12.79	0.85	
496.339	7.03	8.94	LysoPC(16:0)
496.339	7.12	7.83	LysoPC(16:0)
520.508	1.10	2.86	
521.512	1.09	2.95	
538.518	1.10	3.06	Ceramide (d18:1/16:0)

Negative ion mode

m/z	retention time (min)	fold change (allergy/normal)	candidate name $[M-H]^-$
112.988	3.28	1.28	
113.025	9.63	1.43	
124.008	9.63	1.38	taurine
126.003	9.60	1.41	
128.960	10.19	1.15	
130.958	10.45	1.17	
135.030	10.91	0.72	$C_4H_8O_5$
160.062	12.76	1.27	proline +$HCOO^-$
173.009	9.71	1.55	aconitate
173.009	11.84	2.89	aconitate
173.009	12.24	1.84	aconitate
194.1035	9.68	0.59	
196.9471	10.27	1.11	
234.9138	15.52	1.23	
253.217	1.14	3.43	FA[C16:1]
255.233	1.14	6.42	FA[C16:0]
271.057	13.27	0.69	
274.8721	13.11	2.03	
277.218	1.13	1.71	FA[C18:3]
279.233	1.13	1.90	FA[C18:2]
280.236	1.13	1.90	FA[C18:2 (^{13}C)]
281.249	1.12	2.42	FA[C18:1]
283.243	1.12	3.09	
283.265	1.12	3.50	FA[C18:0]
285.752	1.96	1.21	
289.068	13.27	0.70	uridine +$HCOO^-$
301.217	1.13	3.75	FA[C20:5]
303.233	1.12	2.07	FA[C20:4]
304.071	9.54	1.69	
305.240	1.12	2.06	FA[C20:4 (+2^{13}C)]
305.249	1.12	2.67	FA[C20:3]
307.264	1.11	2.95	FA[C20:2]
309.280	1.11	3.45	FA[C20:1]
313.931	1.47	0.76	
317.212	1.18	2.27	FA[C20:5]
321.083	11.08	0.89	
321.244	1.16	2.40	FA[C20:3 (+OH)]
325.114	12.11	0.63	
327.233	1.11	3.25	FA[C22:6]
329.240	1.11	3.01	
329.248	1.11	3.49	FA[C22:5]
330.252	1.11	3.55	FA[C22:5 (+^{13}C)]
331.264	1.11	2.31	FA[C22:4]
335.223	1.21	1.38	FA[C20:4 (+OOH)]
341.212	1.15	1.73	$C_{22}H_{30}O_3$
345.244	1.16	2.63	
361.239	1.19	1.72	FA[C22:5(+2OH)]
375.275	1.14	3.33	MG(16:0/0:0/0:0) +$HCOO^-$
390.122	12.01	1.01	
393.278	1.14	0.70	MG(18:0/0:0/0:0) +CL^-
403.307	1.13	1.19	MG(18:0/0:0/0:0) +HCOO-
404.310	1.13	1.22	
413.094	12.48	0.53	
443.141	12.88	0.55	
484.871	10.16	0.79	

Welch's t-test was performed with adjusted Bonferroni's correction and differential peaks of $p<0.05$ were selected and visualized using Multi Experiment Viewer (MeV) v. Thirty-four peaks among 936 in positive ion mode and 55 among 640 in negative ion mode. Candidate name speculated from accurate mass of the peaks were noted. Many peaks which correspond to the mass of fatty acids were detected such as FA[C18:0], fatty acid with 18 carbons and no double bond. 'HCOO-' is an adduct of formic acid.

Supplementary Table 2: Diet composition of normal diet and special diet given to HR-1 hairless mice

	**Normal Diet**
Ingredients	
Protein	
Casein, Lactic, 30 Mesh	20%
Cystine, L	0.3%
Carbohydrate	
Starch, Corn	39.75%
Lodex 10	13.2%
Sucrose, Fine Granulated	10%
Fiber	
Solka Floc, FCC200	5%
Fat	
Soybean Oil, USP	7%
Minerals	
Calcium Carbonate, Light, USP	1.6%
Potassium Phosphate, Monobasic	0.88%
Sodium Chloride	0.33%
Potassium Citrate, Monohydrate	0.318 %
Potassium Sulfate	0.20%
Magnesium Oxide, Heavy, DC USP	0.10%
Ferric Citrate	0.027%
Zinc Carbonate	0.007%
Sodium Metasilicate	0.006%
Manganese Carbonate Hydrate	0.002%
Copper Carbonate	0.001%
Chromium Potassium Sulfate	0.001%
Boric Acid	<0.001%
Sodium Fluoride	<0.001%
Nickel (II) Carbonate	<0.001%
Lithium Chloride, anhydrous	<0.001%
Sodium Selenate	<0.001%
Potassium Iodate	<0.001%
Ammonium Molybdate Tetrahydrate	<0.001%
Ammonium (meta)vanadate	<0.001%
Vitamins	
Vitamin E Acetate, 50%	0.53%
Niacin (a.k.a. B3)	0.10%
Vitamin B12, 0.1% Mannitol	0.09%
Biotin, 1%	0.07%
Pantothenic Acid, d, Calcium (a.k.a. B5)	0.05%
Vitamin D3, 100,000 IU/gm	0.03%
Vitamin A Acetate, 500,000 IU/gm	0.03%
Pyridoxine HCl (a.k.a. B6)	0.03%
Riboflavin (a.k.a. B2)	0.02%
Thiamine HCl (a.k.a. B1)	0.02%
Folic Acid	0.007%
Phylloquinone (a.k.a. Vitamin K1)	0.003%
Choline Bitartrate	0.25%
Anti-oxidents	
tert-Butylhydroquinone (tBHQ)	0.001%

Normal diet (D10012G) was purchased from research diet, Inc. *For high saturated fatty acid diet and high unsaturated fatty acid diet, powdered chow was added with 8% (w/w) of palmitic acid (PA, Tokyo chemical Industry Co. Ltd) or 8% (w/w) oleic acid (OA, Tokyo chemical Industry Co. Ltd), respectively.
